# Systematic genome sequence differences among leaf cells within individual trees

**DOI:** 10.1186/1471-2164-15-142

**Published:** 2014-02-19

**Authors:** Deepti Diwan, Shun Komazaki, Miho Suzuki, Naoto Nemoto, Takuyo Aita, Akiko Satake, Koichi Nishigaki

**Affiliations:** 1 Graduate School of Science and Engineering, Department of Functional Materials Science, Saitama University, Saitama 338-8570, Japan; 2 Department of Science, Hokkaido University, Sapporo, Japan; 3 Graduate School of Information Science and Technology, Osaka University, Suita, Japan

**Keywords:** Genome sequence, Genomic distance, Mutation rate, Japanese beech, Yoshino cherry, Leaf genomes, Genome profiling (GP), Next-generation sequencing (NGS)

## Abstract

**Background:**

Even in the age of next-generation sequencing (NGS), it has been unclear whether or not cells within a single organism have *systematically* distinctive genomes. Resolving this question, one of the most basic biological problems associated with DNA mutation rates, can assist efforts to elucidate essential mechanisms of cancer.

**Results:**

Using genome profiling (GP), we detected considerable systematic variation in genome sequences among cells in individual woody plants. The degree of genome sequence difference (genomic distance) varied *systematically* from the bottom to the top of the plant, such that the greatest divergence was observed between leaf genomes from uppermost branches and the remainder of the tree. This systematic variation was observed within both Yoshino cherry and Japanese beech trees.

**Conclusions:**

As measured by GP, the genomic distance between two cells within an individual organism was non-negligible, and was correlated with physical distance (i.e., branch-to-branch distance). This phenomenon was assumed to be the result of accumulation of mutations from each cell division, implying that the degree of divergence is proportional to the number of generations separating the two cells.

## Background

At the beginning of the 21st century, genome sequences of two closely related species, human and chimpanzee, were found to differ by approximately 4% based on conventional genome sequencing technology [1]. With the advent of next-generation sequencing (NGS), it has been established that each person has a unique genome [2]. Within a single organism, genome sequences may be epigenetically different between cells, and sporadic differences are sometimes present between cells from different organs [3]. It is not clear, however, whether each cell within an individual organism possesses a *systematically* different genome sequence.

Various breakthroughs have been steadily reshaping our understanding of genomes. These advances include accumulating analyses of whole-genome sequences of individuals [4,5], identification of various non-coding RNAs [6], discovery of the existence of highly repeated sequences [7], and recognition of frequent recombination of genome structures [8,9]. Recently, an intensive study on the fate of cancerous cells by NGS revealed that lineages of such cells are vigorously mutating [10]. Advanced papers on this topic have subsequently appeared [11,12].

**Figure 1:**
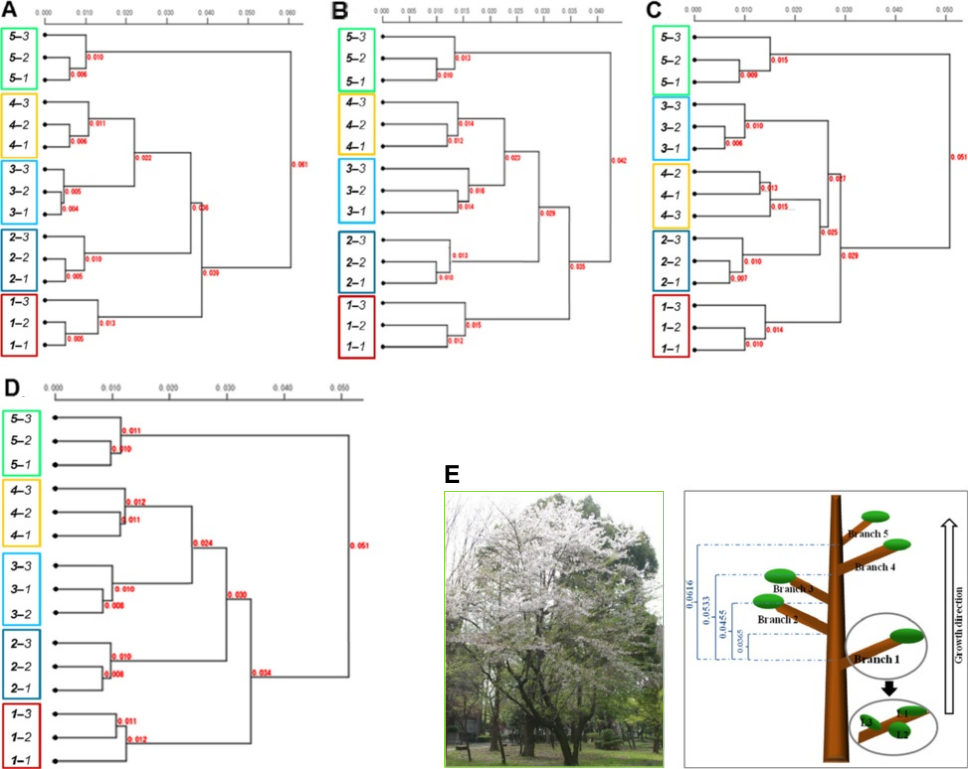
**Clustering of Yoshino cherry tree leaves. (A–C)** Dendrograms resulting from Ward’s cluster analysis of genomic distances of leaves from five tree branches of a Yoshino cherry tree. Each analysis used genomic distances calculated from one of three independent GP experimental trials using the same leaves. Genomic distances are displayed on dendrogram branches. **(D)** Dendrogram obtained from global clustering of leaves from the Yoshino cherry tree. Genomic distances analyzed were calculated from averaged spiddos data obtained from three independent GP experimental trials using the same leaves (for details see in Additional file 4: Table S2). **(E)** Yoshino cherry tree from which young leaves were sampled in April 2010, after the flowering season. The tree was located on the campus of Saitama University.

On the other hand, genome sequence differences have been examined by the copy number variation analysis between normal cells within a single organism [13-15], which informed us of frequent occurrence of mutation in the form of replication slippage at particular genomic loci. In a sense, this is a filtered (i.e., restricted to the tandem repeat sequences) observation of genome alterations. More wide observation of normal genomic DNA is just beginning as can be seen in the recent report [3,16]. Our study is the first to detect *systematic* genome sequence differences among cells in single organisms, i.e., within individuals of two woody plant species (Figure 1E).

There has been a hypothesis (genetic mosaicism hypothesis) that long statured plants accumulate spontaneous mutations that expanded among modules (shoots, branches, leaf etc.) and become genetically mosaic as they grow [17]. This hypothesis is explicitly based on the idea of finite spontaneous mutation rate. That is, DNA replication proceeds with limited accuracy, i.e., 10^-6^ to 10^-9^ errors/base/replication [18] and thus every replicated genome sequence (e.g., the 3 × 10^9^-bp sequence of the human genome) naturally differs from its parental genome. In general, these differences were too small to be directly detected, as they were often below the detection limit of sequencing analysis. Consequently, mutation rate has been conventionally estimated indirectly based on phenotypic changes, such as variation in antibiotic resistance. This situation has been changed by the advent of the NGS (next generation sequencing), enabling the detection of low rate of mutations [19]. However, its application is limited mainly due to high cost and difficulty in data processing [20].

**Figure 2:**
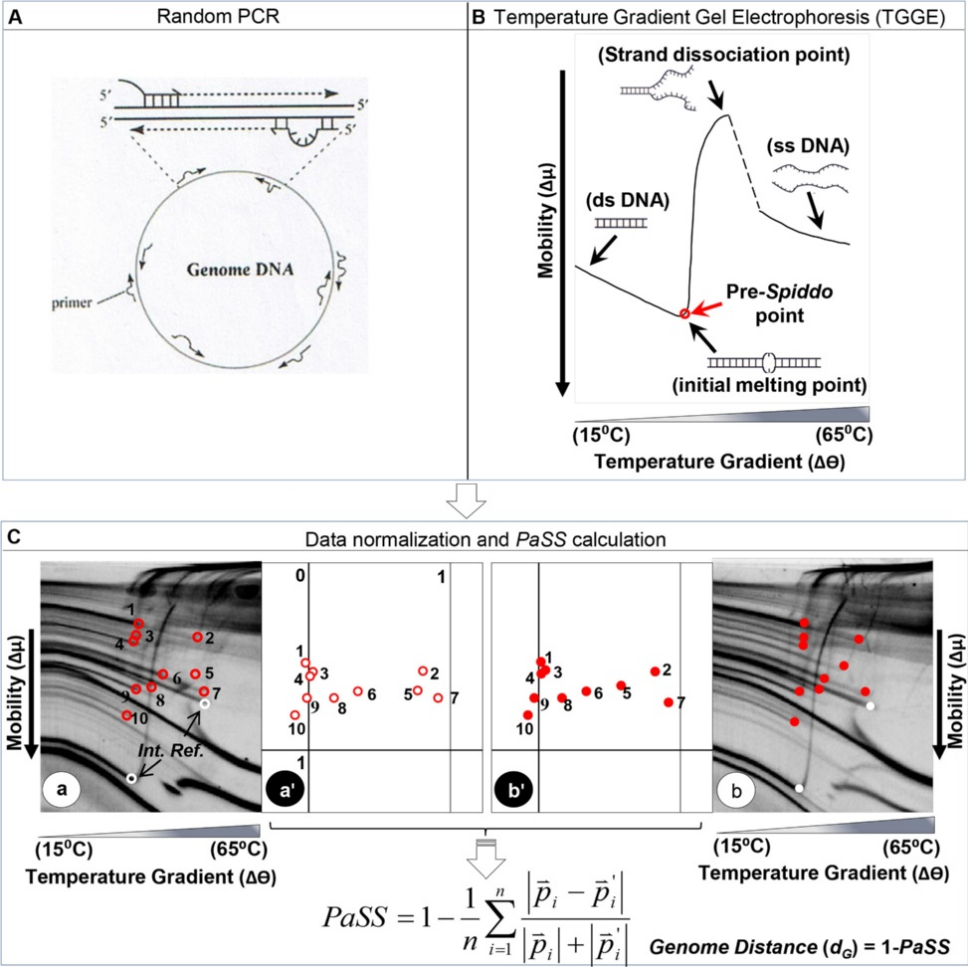
**Overview of the Genome Profiling (GP) method.** The entire GP process consists of three steps: **(A)** Random sampling of DNA fragments from genomic DNA (i.e., random PCR), **(B)** acquisition of sequence information without sequencing (i.e., μTGGE analysis), and **(C)** computer-aided conversion of raw data to genome-intrinsic parameters (spiddos). **(A)** In Random PCR, primers bind to various regions of genomic DNA with mismatch-containing structures under low stringency conditions, leading to the generation of a set of fragments. **(B)** In μTGGE, DNA fragments loaded at the top of a slab gel migrate downward with a characteristic curvature caused by the temperature gradient. The pre**-**spiddo point of a DNA fragment (i.e., initiation of the melting-derived transition from double-stranded to single-stranded DNA) is indicated by a red dot. **(C)** Pre-spiddo points (red dots) are indicated in images **a** and **b** for genomes **a** and **b**, respectively. Species identification dots (spiddos), shown in diagrams **a'** and **b'**, are obtained by normalizing the coordinates of pre-spiddo points with respect to internal reference DNA fragments (white dots). Spiddos thus obtained are used to calculate pattern similarity score (*PaSS*) or genomic distance (*d*_*G*_ = 1 - *PaSS*).

Fortuitously, Genome Profiling (GP) (Figure 2), an easily operable and informative genome analysis method [21-28] is sufficiently competent to detect differences between closely related cells [27,28]. Compared with conventional sequencing approaches, GP involves two unique procedures (Figure 2): i) collection of DNA fragments from genomic DNA by random PCR [29] and ii) acquisition of DNA sequence information using micro-temperature gradient gel electrophoresis (μTGGE) by separating DNA fragments and observing their melting profiles (Figure 2B) [30-32]. In this method, the property, *spiddos* (species identification dots), derived from the DNA sequence information [22] plays the pivotal role in identifying a genome and enables us to measure the genome distance (see Methods).

GP has been used as a tool for universal species identification [21,24,27,28,33] and as an accurate detector of mutation [34,35]. In this study, we applied the GP method to a new challenge: detection of extremely small genomic differences between very closely related cells with the aim of examining within-organism sequence variation.

## Results and discussion

**Figure 3:**
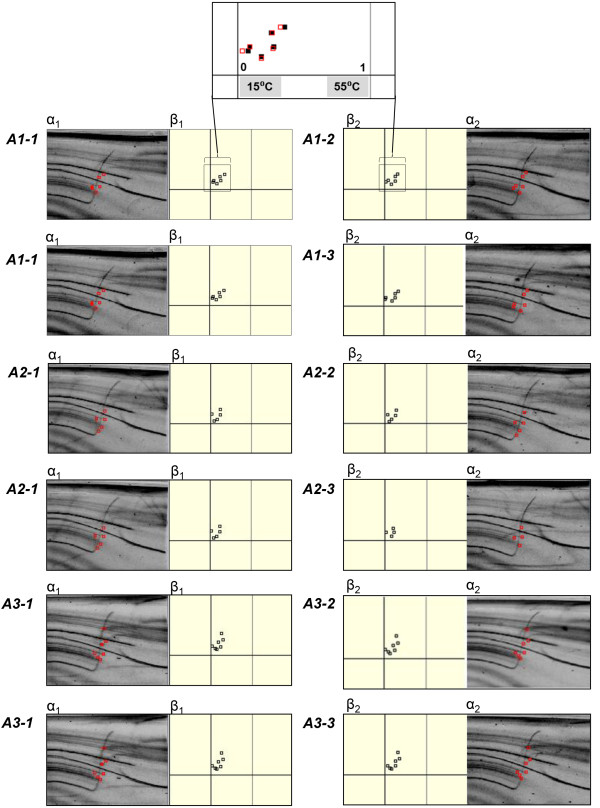
**An example of raw data used for obtaining genomic distance (*d***_***G***_**).** The original data used to obtain Figure 4A (*A1-1* to *A3-3*) are displayed here to demonstrate how *d*_*G*_ values were obtained. Feature points appearing in the genome profiles (TGGE electrophoretic patterns) of two leaves, α_1_ and α_2_ , are indicated by dots. These were processed to provide normalized coordinate data referred to as spiddos (shown in β_1_ and β_2_). The computer-processed data (spiddos) from β_1_ and β_2_ are superimposed so that differences in the two sets of spiddos can be easily recognized. To calculate *PaSS* (defined in Methods), the displacements were summed and divided by the number of spiddos.

**Figure 4:**
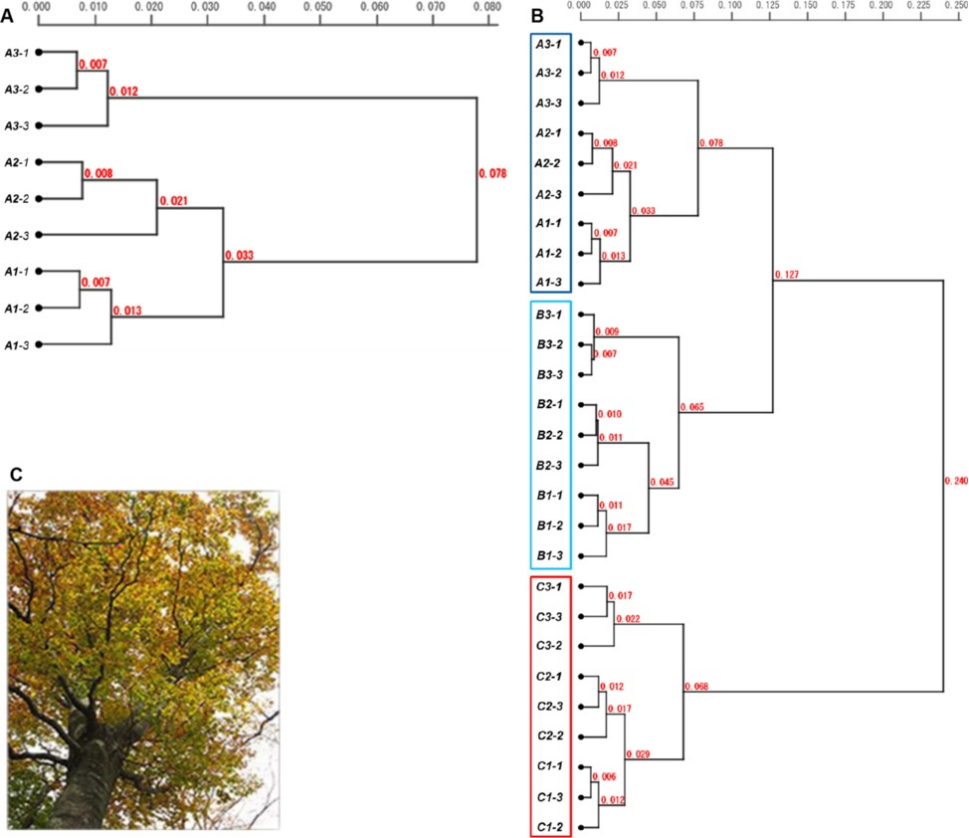
**Clustering of beech tree leaves.** Sample labels indicate the tree, branch, and leaf (e.g., *A1-2* corresponds to leaf *2* of branch *1* of tree *A*). **(A)** Dendrogram resulting from Ward’s clustering of genomic distances of Japanese beech tree leaves. Genomic distances are displayed on dendrogram branches. **(B)** Dendrogram obtained from cluster analysis collectively performed on three different Japanese beech trees. Leaves belonging to each tree clustered together in a fashion similar to the dendrogram shown in **A** even in this global clustering. Each spiddos data point used to calculate genomic distance represented the average of two trials using the same leaf (Additional file 3: Table S1). **(C)** One of the beech trees sampled in Sapporo, Japan in late May, 2011.

We used Japanese beech (*Fagus crenata*) trees to examine whether GP was able to reveal if all leaves within a single tree had identical genome sequences (Figures 2 and 3). More specifically, we analyzed sets of **sp**ecies **id**entification **do**ts (spiddos), a pivotal GP parameter derived from genome sequences (Figure 2C), that were obtained from genome profiles, specified by both mobility and melting temperature, both of which are determined after calibration and normalization of band patterns by a computer using co-migrating internal references (see Methods). Although genome profiles (i.e., DNA patterns generated by μTGGE analysis) were not always reproducible because of experimental fluctuations (i.e., environmental temperature, instrumental drift and others), spiddos were highly reproducible as a result of a normalization process that compensated for experimental fluctuations (Figure 2). As shown in Figure 4A, all leaves on the same Japanese beech branch (e.g., *A1-1*, *A1-2*, and *A1-3,* where *“A1-2”* refers to tree *A*, branch *1*, leaf *2*) clustered together. This was also the case for the genome profiles of leaves on branches *A2* and *A3*. Leaves from different branches were found to have different genome sequences. Spiddos of branch *A1* and *A2* leaves were more similar to one another than to spiddos of leaves on branch *A3*, located furthest from the ground (Figure 3). Differences were observed in spiddos between leaves belonging to the same branch, but these differences were the level of experimental errors and thus they cannot be said to be significant at this moment [22]. These results reveal that within statistical significance, leaves from individual branches possessed identical genome sequences, but had distinctively different sequences from those of different branches, a finding not previously reported. This result was further confirmed by conducting a similar experiment using different Japanese beech individuals. We also analyzed another species, Yoshino cherry (*Prunus* × *yedoensis*), located ~800 km from the site of the Japanese beech trees for more generalized confirmation (Figure 1). Finally, to detect methodological differences, we sequenced a particular DNA band obtained from GP (see in Additional file 1: Figure S1 and for details see Additional file 2). Throughout these experiments, we consistently reached the same conclusion: genome sequences within organisms were not identical, but instead varied *systematically*.

Figure 4B reveals that very similar results were obtained from the two additional Japanese beech trees. Interestingly, the same relationship trend was observed among all three trees: spiddos of leaves from uppermost branches (*A3*, *B3*, and *C3*) were distinct from spiddos of other leaves (Figure 4B). The cluster dendrogram in Figure 4B was globally constructed based on the whole set of distances (*d*_*G*_) obtained from all leaf spiddos (Additional file 3: Table S1); consequently, the resulting logically expected structure—leaves on the same branch grouped together and branches on the same tree clustered together—is most impressive and unexpected, demonstrating the effectiveness of this approach. It is therefore evident that genomes of leaves on a tree are neither completely identical to one another nor randomly different but, rather, systematically differ depending on branch location.

As shown in Figure 1, similar results were reproducibly obtained using the other species, Yoshino cherry. Results of cluster analyses of distances (*d*_*G*_) obtained using spiddos data from three independent GP experiments using the same samples from five branches (Additional file 4: Table S2) are shown in Figure 1A-C; clustering results based on an average of the three trials are shown in Figure 1D. These results of individual experiments (Figure 1A,C) show basically the same pattern as those obtained from the statistically more reliable averages (Figure 1D), indicating that this experimental system has a rather low variance (in other words, a single experiment can provide a good prospect) with only a minor exception: positional exchange of branches *3* and *4* in Figure 1C. The situation observed in Figure 4 (Japanese beech) also held true for Yoshino cherry, i.e., genome profiles of leaves were not identical, but instead differed systematically. In addition, genomes of leaves from the uppermost branch (*5*–*1*, *5*–*2*, and *5*–*3*) were genetically distant from leaves of middle branches, indicating a correlation between genomic distance and branch location. The same phenomenon was thus observed in two different, widely separated species, namely, that leaves from the same tree have different genome sequences that can be distinguished using GP.

Our discovery was partially corroborated upon further investigation using direct sequencing. As shown in (see Additional file 1: Figure S1), leaves from the same branch tended to have more closely related sequences, as seen in pairs of leaves from the same Japanese beech branches (*B2-2* and *B2-3*) and (*B3-1* and *B3-2*) in (see Additional file 1: Figure S1B) and from closely located branches of Yoshino cherry (*B2-1* and *B3-1*) and (*B4-1* and *B5-1*) in (see Additional file 1: Figure S1A). Because of missing data caused by generation of artifacts during cloning and sequencing, these results are somewhat equivocal; nonetheless, these data are congruent with the conclusions drawn from the GP experiments. With respect to these direct sequencing results, the experimental procedures used, and sequencing in general, need to be taken into account. DNA fragments generated from the GP experiment were collected by excising their bands from polyacrylamide gels, the most reliable method for obtaining sequences common to both GP and conventional sequencing. Collected DNA was then subjected to cloning and sequencing, two procedures that can introduce mutations. Many spurious sequences were in fact obtained and discarded, including sequences having very low sequence similarity to the primary sequence generated from the DNA band, and sequences of non-plant origin. Although they were within an apparently acceptable range based on sequence consistency (i.e., high similarity), the results shown in (see Additional file 1: Figure S1) were thus subject to limitations inherent to the cloning and sequencing process. Nevertheless, this illustrates one difficulty encountered when using such a clone-isolation- and sequencing-based approach to identify mutation frequencies: the two mutation types—original mutations and sequencing operation-derived mutations (presumably introduced during template preparation, PCR-amplification, sequencing, and base-calling), cannot be distinguished in the final clonal sequencing results. To obtain statistically significant results using conventional high-precision sequencing, high-volume sequencing of the multiple-million base-pair level must be carried out to separate infrequently occurring mutations (e.g., < 10^-6^/mutations/base/replication) from background noise. In this regard, it should be noted that the ability of the GP method to overcome this difficulty has been experimentally demonstrated: GP has been used successfully for species identification and classification [24,25,27,28,36] and in high-sensitivity mutation assays [34,35].

**Figure 5:**
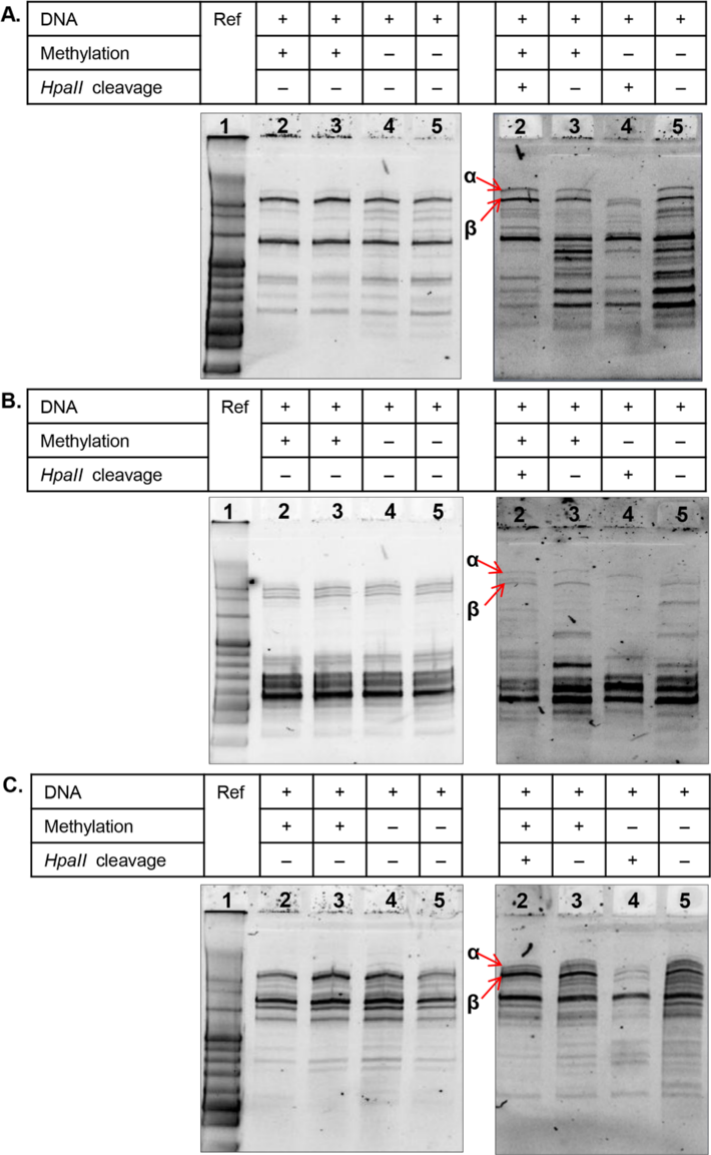
**DNA replication is not affected by DNA methylation.** As shown in Panels **A**, **B**, and **C**, the results of three independent tests using different portions of yeast genomic DNA (which is naturally unmethylated [38]) provide evidence that methylation does not affect PCR results. In these experiments, random PCR was performed using one of the primers (pfm 3 (5'-cy3-dCTGGATAGCGTC), pfm 10 (5'-cy3-dGCGCATTAGACG) and pfm 12 (5'-cy3-dAGAACGCGCCTG)) with *Taq* DNA polymerase. (Random PCR is a variation of PCR employing only a single primer and performed at a lower annealing temperature [~26°C], generating primer sequence-independent DNA fragments [31]). Lane 1 is a100-bp size marker. Bands indicated by α and β (in lane 2 of panels **A**, **B** and **C**) are DNA fragments containing *HpaII* methylation/restriction site(s), as their cleavage resulted in their disappearance from lane 4. The presence of α and β bands in lane 2 in panels **A**, **B** and **C** demonstrate that these regions could be amplified by random PCR even though they contained a methylation site.

In this study, we have demonstrated that leaves from the same tree do not have exactly identical genome sequences. This conclusion is expected to be applicable to any multi-celled organism, as DNA is not perfectly replicated in any organism, and thus each genome replication cycle induces mutations that are usually too infrequent to be detected (10^-6^ to 10^-9^ mutations/base/replication) [18]. In addition, epigenetic methylation of DNA, of which degree must be different from cell to cell and may have a potential to induce base-substitution during PCR, does not effect its PCR amplification [37], which was independently confirmed in our study (Table 1 and Figure 5). Based on the total number of base pairs in the DNA bands obtained by random PCR (i.e., roughly 10 bands, each 1000 bp), we tentatively estimate the GP method has a detection sensitivity of 10^-4^ mutations/base/replication. More specifically, the total number of mutations accumulating over *g* generations, *μ(g)*, can be calculated using the formula:

(1) $$\mu \left(g\right)=\sum_{i=1}^{g}\left(\mu \left(i\right)+\gamma \left(i\right)\right),$$

where *μ(i)* and *γ(i)* represent replication-dependent and repair-dependent mutation rates, respectively. If we tentatively assume *μ(i) = μ*_*c*_ (a constant) and *μ(i) > > γ(i)* for all *i*, then

(2) $$\mu \;\left(g\right)=g.\mu_{c}$$

**Table 1:** Reagents used for the DNA methylation and restriction enzyme cleavage

**Step 1. Methylation reaction (10 μl) from the protocol of New England Biolabs Inc.**	
Nuclease free water	6 μl
10× HpaII methyltransferase buffer	1 μl
SAM (80 μM)	0.1 μl
Genomic DNA (10 ng)	0.4 μl
*HpaII* methyltransferase	2.5 μl
**Step 2. *HpaII *** **digestion reaction (50 μl)**	
Methylation product (taken from step 1)	10 μl
10× NEBuffer 1	4 μl
MgCl_2_ (10 mM)	20 μl
*HpaII* restriction enzyme	4 μl
Nuclease free water	12 μl

SAM; S-adenosylmethionine, NEBuffer; New England Biolabs buffer.

This estimate indicates that the GP method cannot detect mutations occurring at a frequency lower than g *· μ*_*c*_ (*≤* 10^-4^/base). Consequently, leaf genomes must contain a significant number of mutations, equivalent to the sum of replication- and repair-caused mutations. This finding leads us to consider whether the large number of estimated mutations implies that mutation events during replication and repair (a type of 'somatic mutation’) have been unexpectedly frequent [39], or if instead there is a large cell generation difference between tree branches, as follows:

If we assume that *μ*_*c*_ = 10^-8^ in the above context, then *g*, the number of generations, must be

(3) $$g=\frac{\mu \left(g\right)}{\mu_{c}}\underline{\underline{>}}\frac{10^{-4}}{10^{-8}}=10^{4}$$

Because longitudinally tandem consecutive cells expand to the length *g' · a*, where *g'* is the number of cell generations and *a* is the unit cell length, we can calculate the number of cell generations (*g'*) separating two branches. If *a* = 20 μm and the branch-to-branch distance, *B*, is 2 m, then

(4) g′=Ba=22×10-5=105>g=104

and thus from Eq. 2,

(5) $$\mu_{c}=\frac{\mu \left(g\right)}{g}>\frac{\mu \left(g\right)}{g^{\prime }}=\frac{10^{-4}}{10^{5}}=10^{-9}$$

Based on this tentative calculation, the apparent genomic distance observed using the GP method, which has a detection limit ≥ 10^-4^/base, is within a reasonable range. In other words, the accumulated point mutations are as a consequence of the large generational difference between cells. Obviously, this conclusion needs to be confirmed by other approaches. Our finding regarding this unexpectedly wide genome-to-genome distance will surely collect the interest in this theme which have been less payed with attention.

Except for cancer cells, cells within an individual organism have been previously believed to possess identical genomes. Two brief reports have recently appeared suggesting that cells from a single individual might have different genomes [3,16], although no hard evidence exists nor has systematic research been performed to confirm those observations. Nevertheless, these reports are consistent with the findings of our study.

## Conclusions

The study reported here provided with the first *systematic* analysis of genome sequence differences among cells in single individuals using the GP method. As a result leaf genome sequences within individual trees were found not to be identical, but varied *systematically* from the bottom to the top of the tree. Since this phenomenon was detected by the GP method that cannot detect the mutation of less than 10^-5^/base/replication, a large number of accumulated mutations must exist between distantly located cells in the tree.

This fact leads to a natural inference that two cells in an individual differ in their genome sequences in relation to their physical distances. In other words, no two cells have completely identical genome sequences. This finding and inference will surely have an influence on the interpretation of various phenomena including mutagens, cancer and others.

## Methods

Leaves of Japanese beech (or Buna) (*Fagus crenata*) trees growing in Sapporo, Japan, and Yoshino cherry (Sakura) (*Prunus* × *yedoensis*) trees from Saitama, Japan, were used in this study. The notation *A1-2* denotes leaf *2* on branch *1* of tree *A*. Branch numbers were assigned in the order in which they appeared, beginning from lower (ground) to upper (tree top) levels.

### Genomic DNA preparation

After washing leaf samples in 10% sodium dodecyl sulfate (SDS), DNA was extracted using the cetyltrimethylammonium bromide (CTAB) method [40]. Briefly, 100–120-mg samples (wet weight) were homogenized with a mortar and pestle using liquid nitrogen. One milliliter of CTAB solution (200 mM Tris–HCl [pH 9.0], 2% [w/v] CTAB, 2% [w/v] polyvinylpyrrolidone, 0.1% [v/v] 2-mercaptoethanol, 1.4 M NaCl, and 20 mM ethylenediaminetetraacetic acid) was immediately added to the crushed cells, followed by incubation for 1 h at 65°C. After incubation, a 24:1 chloroform-isoamyl alcohol mixture was added; the solution was mixed gently and then centrifuged for 10 min at 12,000 × *g* (14,000 rpm). This step was repeated twice. An equal volume of propanol was then added to the supernatant, which was centrifuged for 5 min at 16,000 × g (15,000 rpm). In most cases, the pellet obtained was washed with 70% ethanol, centrifuged, and desiccated using an evaporator. Finally, 100 μl of phosphate-buffered saline was added to the precipitate to dissolve the pellet.

GP technology is sufficiently robust such that slight impurities of denatured proteins or polysaccharides will not interfere. Other plant cell components, such as alkaloids and secondary products, can be inhibitory to the PCR reaction, however; consequently, DNA samples were diluted prior to amplification.

### Genome profiling (GP)

Genome profiling (GP) uses a set of DNA fragments sampled from genomic DNAs, and is composed of three fundamental steps: random PCR, micro-temperature gradient gel electrophoresis (μTGGE), and data normalization by computer processing [22,32] (Figure 2). Random PCR can employ arbitrary primers for the PCR reaction because of the relaxed nature of primer binding to template DNA under sufficiently low temperatures. This attribute allows samples of unknown genomic sequence, for which specific primers cannot be designed, to be amplified. As a consequence, DNA fragments from any genomic DNA can be collected independently of the sequence of an oligonucleotide primer used [30,31] (Note that a single primer is used for random PCR).

### Random PCR

Random PCR was performed using primers HUNT (5′-dTGCTGCTGCTGC-3′) and Pfm12 (5′-dAGAACGCGCCTG-3′), which were Cy3-labeled at their 5′ ends. The reaction mixture (25 μl total volume) for random PCR contained 1 ng template DNA, 100 μM primer DNA, 200 μM dNTPs, 10 mM Tris–HCl (pH 9.0), 50 mM KCl, 2.5 mM MgCl_2_, and 0.02 unit μl^-1^*Taq* DNA polymerase (Takara Bio Inc., Shiga, Japan). During random PCR, contamination by other organisms should be carefully avoided. To inactivate any contaminating DNAs that could act as a template, the entire random PCR solution, without the template DNA, was therefore UV-irradiated prior to the reaction. Random PCR was carried out using 30 cycles of denaturation (94°C, 30 s), annealing (26°C, 1 min), and extension (47°C, 1 min) on a C1000 thermal cycler (Bio-Rad, Hercules, CA, USA). The second random PCR mixture (50 μl volume) contained 1 μl of the first PCR product as template and the same concentrations of constituents used in the original reaction. The reaction was performed using 10 cycles of denaturation (94°C, 30 s), annealing (60°C, 1 min), and extension (74°C, 1 min). Only 10 cycles were used to ensure that the reaction was terminated before all primer molecules were consumed; this was necessary to guarantee that the major PCR products were in a double-stranded state and thus suitable for TGGE analysis (i.e., so that the melting transition of double-stranded DNA to a single-stranded form can be detected).

### μTGGE analysis

For μTGGE, we used a tiny slab gel (24 × 16 × 1 mm^3^) set on a μ-TG temperature-gradient generator (Taitec, Iruma, Japan) for electrophoresis [32]. Two internal reference DNAs with known melting patterns were co-migrated during each electrophoretic run to calibrate each genome profile, giving highly reproducible results [41]: a 200-bp Ref1 (a 191-bp fragment from the bacteriophage fd gene VIII, sites 1350–1540, attached to a 9-bp sequence, CTACGTCTC, at the 3′ end; T_m_ = 60°C) and a 900-bp Ref2 taken from a 4361-bp pBR322 fragment (T_m_ = 61.4°C). Fluorescently-labeled primers MA1 (5′-cy3-dTGCTACGTCTCTTCCGATGCTGTCTTTCGCT-3′) and MA2 (5′-dCCTTGAATTCTATCGGTTTATCA-3′), Ref6F (5′-cy3-dGCCGGCATCACCGGCGCCACAGGTGCGGTTG-3′), and Ref6R (5′-dTAGCGAGGTGCCGCCGGCTTCCATTCAGGTC-3′) were used to generate internal references 1 and 2, respectively. The gel used was 6% polyacrylamide (19:1 acrylamide:bis) containing 500 mM Tris–HCl, 485 mM boric acid, 20 mM EDTA (pH 8.0), and 8 M urea. Approximately 2 μg of DNA was loaded onto the gel and subjected to electrophoresis with a linear temperature gradient of 15 to 65°C for 12 minutes at 100 V cm^-1^. After electrophoresis, DNA bands were detected using an FX Molecular Imager fluorescence imager (Bio-Rad).

### Computer-aided data analysis

Genome profiles obtained by the GP method are highly informative, but difficult to interpret because of their complexity. To overcome this problem, feature points called spiddos can be introduced [22]. Spiddos correspond to points where DNA structural transitions occur, such as from double-stranded to single-stranded DNA [42]. The coordinates of *spiddos* are established to be reproducibly obtained by an internal reference-mediated normalization (i.e., the coordinates of the two reference points contained in each GP profile (ref 1 and ref 2, Figure 2C) are used to calibrate the coordinates of the featuring points for same DNAs) which is sequence- and size-dependent.

Using these normalized coordinates, a pattern similarity score (*PaSS*) between two genomes can be measured as follows:

(6) $$\mathrm{PaSS}=1-\frac{1}{n}\sum_{i=1}^{n}\frac{\left|{\vec{p}}_{i}-{\vec{p}}_{i}^{'}\right|}{\left|{\vec{p}}_{i}\right|+\left|{\vec{p}}_{i}^{'}\right|},$$

where ${\vec{p}}_{i}$ and ${\vec{p}}_{i}^{'}$ correspond to the normalized positional vectors (composed of two elements: mobility *μ* and temperature θ) for spiddos ${\vec{p}}_{i}$ and ${\vec{p}}_{i}^{'}$ collected from two genome profiles, respectively, and *i* denotes the spiddo serial number. In general, 0 ≤ *PaSS* ≤ 1, and thus, 0 ≤ *d*_*G*_ ≤ 1. *PaSS* is equal to one when two spiddo sets match perfectly*.*

Genomic distance (*d*_*G*_), a more practical form, is derived from *PaSS* as follows:

(7) $$d_{G}=1-\mathrm{PaSS}$$

If *d*_*G*_ is sufficiently small (<< 1), the two genomes of interest belong to the same species.

### Cluster analysis of GP data

To cluster species based on calculated *d*_*G*_ values, we used Ward’s clustering method as implemented in the software program FreeLighter [25,43,44].

### Sequencing

DNA bands of interest were extracted from TGGE microgels and used as PCR templates in reaction mixtures containing 320 μM dNTPs, 100 μM primer pfm12 (5′-dAGAACGCGCCTG-3′), 10 mM Tris–HCl (pH 9.0), 50 mM KCl, 2.5 mM MgCl_2_, and 0.02 unit μl^-1^*Taq* DNA polymerase (Takara). Reaction conditions consisted of 30 cycles of denaturation at 94°C for 30 s, annealing at 60°C for 60 s, and extension at 74°C for 60 s. The resulting random PCR products (DNA) were ligated to pGEM-T Easy vectors (Promega, Madison, WI, USA) at 4°C overnight. Competent cells of *E. coli* DH5α (Toyobo Co. Ltd., Osaka, Japan) were transformed with the ligation product. Transformed cells were cultivated on LB agar plates (1% tryptone, 0.5% yeast extract, 1% NaCl [pH 7.0], and 1.5% agar) supplemented with ampicillin (10 mg in 200 ml of LB media), 20 μl X-Gal (50 mg ml^-1^ in dimethyformamide), and 100 μl of 0.1 M IPTG (isopropylthio-β-galactoside). The agar plates were incubated at 37°C for 12–14 h. White colonies on the plates were selected with a sterile toothpick, transferred to LB broth (1% tryptone, 0.5% yeast extract, and 1% NaCl, pH 7.0; 10 mg ampicillin), and incubated at 37°C for 12–14 h with shaking at about 180 rpm. After confirmation of gene insertion, plasmid DNA was purified using a Wizard Plus SV Minipreps DNA purification system (Promega) and commercially sequenced (Operon Bio-technology, Tokyo, Japan).

### Availability of supporting data

Data sets supporting the results of this study are included within the article and its additional files.

## Abbreviations

GP: Genome profiling; NGS: Next-generation sequencing; μTGGE: Micro-temperature gradient gel electrophoresis; Spiddos: Species identification dots; PaSS: Pattern similarity score; dG: Genomic distance.

## Competing interests

The authors declare that they have no competing interests.

## Authors’ contributions

SK carried out initial experiments to establish the overall experimental protocol, and collected and processed GP data. DD performed GP analyses and supplementary experiments, including methylation experiments, finalized the study, and wrote the manuscript. MS, NN, and TA analyzed the data; AS helped analyze the data and collected samples. KN designed and directed the study, analyzed data, and helped write and edit the manuscript. All authors read and approved the final manuscript.

## Supplementary Material

Additional file 4: Table S2 Genome distances (*d*_*G*_) among genomes of Yoshino Cherry tree leaves. Each value is the average of three independent experiments. Click here for file

Additional file 1: Figure S1 Sequence-based clustering of leaves from (A) Yoshino cherry and (B) Japanese beech trees. Only sequence data that could be consistently assigned were used. Clustering was performed using Consensus Maker v2.0.0 (http://www.hiv.lanl.gov/content/sequence/CONSENSUS/consensus.html). Yoshino cherry tree leaf number designations are arbitrary. Click here for file

Additional file 2 **DNA consensus sequence data of leaves used for analysis were derived using Consensus Maker v2.0.0 (**http://www.hiv.lanl.gov/content/sequence/CONSENSUS/consensus.html), and then used to construct clustering tree. (These sequences are deposited in GenBank database: KJ411230-KJ411277). Click here for file

Additional file 3: Table S1 Genome distances (*d*_*G*_) among genomes of Japanese beech tree leaves. Each value is the average of two independent experiments. Click here for file
