# Supplementary figures and images for: Systematic genome sequence differences among leaf cells within individual trees
Source: BMC Genomics. 2014 Feb 19;15:142. doi: 10.1186/1471-2164-15-142 (PMC3937000; doi:10.1186/1471-2164-15-142)

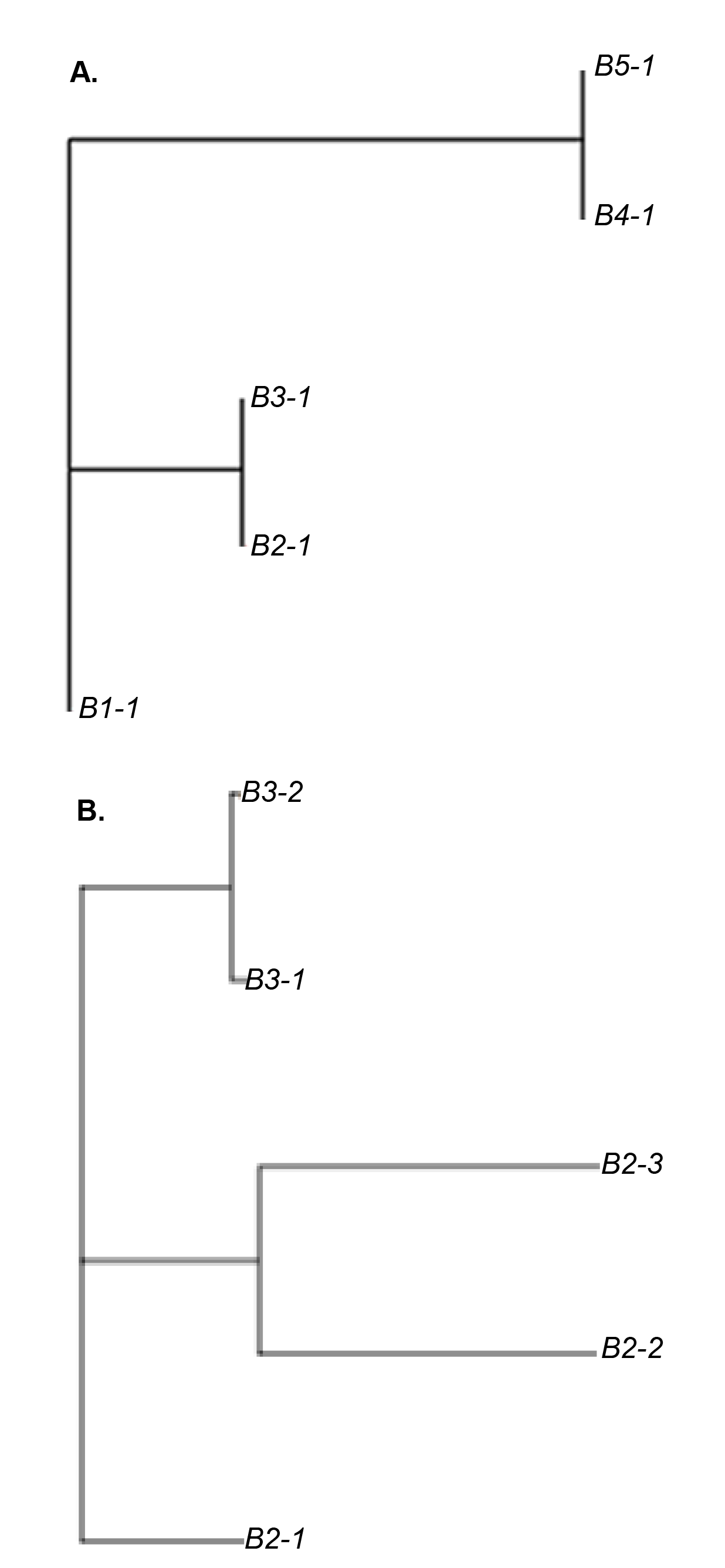

Supplement: Additional file 1: Figure S1 — Sequence-based clustering of leaves from (A) Yoshino cherry and (B) Japanese beech trees. Only sequence data that could be consistently assigned were used. Clustering was performed using Consensus Maker v2.0.0 (http://www.hiv.lanl.gov/content/sequence/CONSENSUS/consensus.html). Yoshino cherry tree leaf number designations are arbitrary. [file 1471-2164-15-142-S1.tiff]
