# Supplementary material for: Systematic genome sequence differences among leaf cells within individual trees
Source: BMC Genomics. 2014 Feb 19;15:142. doi: 10.1186/1471-2164-15-142 (PMC3937000; doi:10.1186/1471-2164-15-142)
Supplement: Additional file 2 — DNA consensus sequence data of leaves used for analysis were derived using Consensus Maker v2.0.0 (http://www.hiv.lanl.gov/content/sequence/CONSENSUS/consensus.html), and then used to construct clustering tree. (These sequences are deposited in GenBank database: KJ411230-KJ411277). [file 1471-2164-15-142-S2.docx]

**DNA sequence data of tree leaves:**

**(GenBank accession number: KJ411230-KJ411277)**

>Seq1 [organism= Prunus x yedoensis] B1-1 denotes leaf 1 on branch B1

GCACAAGGTAGGGGTGGCGAGGACAAGGCCTGACAGGCCATGCCTTTTCACGTGGGGGGCGTTTGATAGCCAACCCGTGGTCATATGGCATGGCTCGAACGTGGCTTATATACTTTTAGGTGTTATAAGCTAACGGATGCGATCATACCAACTCACATGCACCGGATCCCATCAGAACTCCGAAGTTAAGCGAGTTTGGGCGAGAGTAGTACTAGGATGGGTGACCTCCTGGGAAGTCCTCGTGTTGCATCCCTTCTTTTTGCCCTTTTGCCGTTCCAATTGCCGTGGGCGTTTGCTTTCCGTCGTCTTATTCATCTGGCTGTCTTTGTTATTCCACGACCCAGTGACGCCGCGTGACAATCGACGCAAACTACGCTTAAATGCTCCGAGAAAGCCCAGGATTAACGCGAGAATTAAAATCGGAAAATAACATAGTTTCCGTAAATTGTAAAGGCCGGATTTCGGGCTAGTAAACGATGTCGGATGCCCGAAAAAGCCCATGAGTCATGGCAACGCAATGAGTTTTACTCGTGACGCCGTTCTCTTCAAATCGGCAGTTCAGGGGCCCAATTCCGAGGTCGGAGCCCGGATCTGCGGTTTACCCGATTTTCCGTTG

>Seq2 [organism= Prunus x yedoensis] B1-1 denotes leaf 1 on branch B1

GCACAAGGTAGGGGTGGCGAGGACAAGGCCTGACAGGCCATGCCTTTTCACGTGGGGGGGCGTTTGATAGCCAACCCGTGGTCATATGGCATGGCTCGAACGTGGCTTATATACTTTTAGGCGTTATAAGCTAACGGATGCGATCATACCAACTCACATGCACCGGATCCCATCAGAACTCCGAAGTTAAGCGAGTTTGGGCGAGAGTAGTACTAGGATGGGTGACCTCCTGGGAAGTCCTCGTGTTGCATCCCTTCTTTTTGCCCTTTTGCCGTTCCAATTGCCGTGGGCGTTTGCTTTCCGTCGTCTTATTCATCCGGCTGTCTTTGTTATTCCACGACCCAGTGACGCCGCGTGACAATCGACGCAAACTACGCTTAAATGCTCCGAGAAAGCCCAGGATTAACGCGAGAATTAAAATCGGAAAATAACATAGTTTCCGTAAATTGTAAAGGCCGGATTTCGGGCTAGTAAACGATGTCGGATGCCCGAAAAAGCCCATGAGTCATGGCATCGCAATGAGTTTTACTCGTGACGCCGTTCTCTTCAAATCGGCAGTTCAGGGGTCCAATTCCGAGGTCGGAGCCCGGATCTGCGGTTTACCCGATTTTCCGTTG

>Seq3 [organism= Prunus x yedoensis] B1-1 denotes leaf 1 on branch B1

CAACGGAAAATCGGGTAAACCGCAGATCCGGGCTCCGACCTCGGAATTGGACCCCTGAACTGCCGATTTGAAGAGAACGGCGTCACGAGTAAAACTCATTGCGATGCCATGACTCATGGGCTTTTTCGGGCATCCGACATCGTTTACTAGCCCGAAATCCGGCCTTTACAATTTACGGAAACTATGTTATTTTCCGATTTTAATTCTCGCGTTAATCCTGGGCTTTCTCGGAGCATTTAAGCGTAGTTTGCGTCGATTGTCACGCGGCGTCACTGGGTCGTGGAATAACAAAGACAGCCGGATGAATAAGACGACGGAAAGCAAACGCCCACGGCAATTGGAACGGCAAAAGGGCAAAAAGAAGGGATGCAACACGAGGACTTCCCAGGAGGTCACCCATCCTAGTACTACTCTCGCCCAAACTCGCTTAACTTCGGAGTTCTGATGGGATCCGGTGCATGTGAGTTGGTATGATCGCATCCGTTAGCTTATAACGCCTAAAAGTATATAAGCCACGTTCGAGCCATGCCATATGACCACGGGTTGGCTATCAAACGCCCCCCCACGTGAAAAGGCATGGCCTGTCAGGCCTTGTCCTCGCCACCCCTACCTTGTGC

>Seq4 [organism= Prunus x yedoensis] B1-1 denotes leaf 1 on branch B1

CAACGGAAAATCGGGTAAACCGCAGATCCGGGCTCCGACCTCGGAATTGGGCCCCCGAACTGCCGATTTGAAGAGAACGGCGTCACGAGTAAAACTCATTGCGTTGCCATGACTCATGGGCTTTTTCGGGCATCCGACATCGTTTACTAGCCCGAAATCCGGCCTTTACAATTTACGGAAACTATGTTATTTTCCGATTTTAATTCTCGCGTTAATCCTGGGCTTTCTCGGAGCATTTAAGCGTAGTTTGCGTCGATTGTCACGCGGCGTCACTGGGTCGTGGAATAACAAAGACAGCCAGATGAATAAGACGACGGAAAGCAAACGCCCACGGCAATTGGAACGGCAAAAGGGCAAAAATAAGGGATGCAACACGAGGACTTCCCAGGAGGTCACCCATCCTAGTACTACTCTCGCCCAAACTCGCTTAACTTCGGAGTTCTGATGGGATCCGGTGCATGTGAGTTGGTATGATCGCATCCGTTAGCTTATAACGCCTAAAAGTATATAAGCCACGTTCGAGCCATGCCATATGACCACGGGTTGGCTATCAAACGCCCCCCCACGTGAAAAGGCATGGCCTGTCAGGCCTTGTCCTCGCCACCCCTACCTTGTGC

>Seq5 [organism= Prunus x yedoensis] B2-1 denotes leaf 1 on branch B2

CAACGGAAAATCGGGTAAACCGCAGATCCGGGCTCCGACCTCGGAATTGGGCCCCTGAACTGCCGATTTGAAGAGAACGGCGTCACGAGTAAAACTCATTGCGTTGCCATGACTCATGGGCTTTTTCGGGCATCCGACATCGTTTACTAGCCCGAAATCCGGCCTTTACAATTTACGGAAACTATGTTATTTTCCGATTTTAATTCTCGCGTTAATCCTGGGCTTTCTCGGAGCATTTAAGCGTAGTTTGCGTCGATTGTCACGCGGCGTCACTGGGTCGTGGAATAACAAAGACAGCCAGATGAATAAGACGACGGAAAGCAAACGCCCACGGCAATTGGAACGGCAAAAGGGCAAAAAGAAGGGATGCAACACGAGGACTTCCCAGGAGGTCACCCATCCTAGTACTACTCTCGCCTAAACTCGCTTAACTTCGGAGTTCTGATGGGATCCGGTGCATGTGAGTTGGTATGATCGCATCCGTTAGCTTATAACGCCTAAAAGTATATAAGCCACGTTCGAGCCATGCCATATGACCACGGGTTGGCTATCAAACGCCCCCCCACGTGAAAAGGCATGGCCTGTCAGGCCTTGTCCTCGCCACCCCTACCTTGTGC

>Seq6 [organism= Prunus x yedoensis] B2-1 denotes leaf 1 on branch B2

CAACGGAAAATCGGGTAAACCGCAGATCCGGGCTCCGACCTCAGAATTGGGCCCCTGAACCGTCGATTTGAAGAGAACGGCGTCACGAGTAAAACTCATTGCGTTGCCATGACTCACGGGCTTTTTCGGGCATCCGGCATCGTTTACTAGCCCGAAATCCGGCCTTTACAATTTACGGAAACTATGTTATTTTCCGATTTTAATTCTCGCGTTAATCCTGGGCTTTCTAGGAGCATTTAAGCGTAGTTTGCGTCGAGTGTCACGCGGCGTCACTGGGTCGTGGAATAACAAAGACAGCCAGATGAATAAGACGACGGAAAGCAAACGCCCACGGCAATTGGAACGGCAAAAGGGCGAAAAGAAGGGATGCAACACGAGGACTTCCCAGGAGGTCACCCATCCTAGTACTACTCTCGCCCAAACTCACTTAACTTCGGAGTTCTGATGGGATCCGGTGCATGTGAGTTGGTATGATCGCATCCGTTAGCTTATAACGCCTAAAAGTATATAAGCCACGTTCGAGCCATGCCATATGACCACGGGTTGGCTATCAAACGCCCCCCCACGTGAAAAGGCATGGCCTGTCAGGCCTTGTCCTCGCCACCCCTACCTTGTGC

>Seq7 [organism= Prunus x yedoensis] B2-1 denotes leaf 1 on branch B2

GCACAAGGTAGGGGTGGCGAGGACAAGGCCTGACAGGCCATGCCTTTTCACGTGGGGGGGGCGTTTGATAGCCAACCCGTGGTCATATGGCATGGCTCGAACGTGGCTTATATACTTTTAGGCGTTATAAGCTAACGGATGCGATCATACCAACTCACATGCACCGGATCCCATCAGAACTCCGAAGTTAAGCGAGTTTGGGCGAGAGTAGTACTAGGATGGGTGACCTCCTGGGAAGTCCTCGTGTTGCATCCCTTCTTTTTGCCCTTTTGCCGTTCCAATTGCCGTGGGCGTTTGCTTTCCGTCGTCTTATTCATCTGGCTGTCTTTGTTATTCCACGACCCAGTGACGCCGCGTGACAATCGACGCAAACTACGCTTAAATGCTCCGAGAAAGCCCAGGATTAACGCGAGAATTAAAATCGGAAAATAACATAGTTTCCGTAAATTGTAAAGGCCGGATTTCGGGCTAGTAAACGATGTCGGATGCCCGAAAAAGCCCATGAGTCATGGCAACGCAATGAGTTTTACTCGTGACGCCGTTCTCTTCAAATCGGCAGTTCAGGGGCCCAATTCCGAGGTCGGAGCCCGGATCTGCGGTTTACCCGATTTTCCGTTG

>Seq8 [organism= Prunus x yedoensis] B3-1 denotes leaf 1 on branch B3

CAACGGAAAATCGGGTAAACCGCAGATCCGGGCTCCGACCTCGGAATTGGGCCCCTGAACTGCCGATTTGAAGAGAACGGCGTCACGAGTAAAACTCATTGCGTTGCCATGACTCATGGGCTTTTTCGGGCATCCGACATCGTTTACTAGCCCGAAATCCGGCCTTTACAATTTACGGAAACTATGTTATTTTCCGATTTTAATTCTCGCGTTAATCCTGGGCTTTCTCGGAGCATTTAAGCGTAGTTTGCGTCGATTGTCACGCGGCGTCACTGGGTCGTGGAATAACAAAGACAGCCAGATGAATAAGACGACGGAAAGCAAACGCCCACGGCAATTGGAACGGCAAAAGGGCAAAAAGAAGGGATGCAACACGAGGACTTCCCAGGAGGTCACCCATCCTAGTACTACTCTCGCCCAAACTCGCTTAACTTCGGAGTTCTGATGGGATCCGGTGCATGTGAGTTGGTATGATCGCATCCGTTAGCTTATAACGCCTAAAAGTATATAAGCCACGTTCGAGCCATGCCATATGACCACGGGTTGGCTATCAAACGCCCCCCCACGTGAAAAGGCATGGCCTGTCAGGCCTTGTCCTCGCCACCCCTACCTTGTGC

>Seq9 [organism= Prunus x yedoensis] B3-1 denotes leaf 1 on branch B3

CAACGGAAAATCGGGTAAACCGCAGATCCGGGCTCCGACCTCGGAATTGGGCCCCTGAACTGCCGATTTGAAGAGAACGGCGTCACGAGTAAAACTCATTGCGTTGCCATGACTCATGGGCTTTTTCGGGCATCCGACATCGTTTACTAGCCCGAAATCCGGCCTTTACAATTTACGGAAACTATGTTATTTTCCGATTTTAATTCTCGCGTTAATCCTGGGCTTTCTCGGAGCATTTAAGCGTAGTTTGCGTCGATTGTCACGCGGCGTCACTGGGTCGTGGAATAACAAAGACAGCCAGATGAATAAGACGACGGAAAGCAAACGCCCACGGCAATTGGAACGGCAAAAGGGCAAAAATAAGGGATGCAACACGAGGACTTCCCGGGAGGTCACCCATCCTAGTACTACTCTCGCCCAAACTCGCTTAGCTTCGGAGTTCTGATGGGATCCGGTGCATGTGAGTTGGTATGATCGCATCCGTTAGCTTATAACGCCTAAAAGTATATAAGCCACGTTCGAGCCATGCCATATGACCACGGGTTGGCTATCAAACGCCCCCCCACGTGAAAAGGCATGGCCTGTCAGGCCTTGTCCTCGCCACCCCTACCTTGTGC

>Seq10 [organism= Prunus x yedoensis] B3-1 denotes leaf 1 on branch B3

CAACGGAAAATCGGGTAAACCGCAGATCCGGGCTCCGACCTCGGAATTGGGCCCCTGAACTGCCGATTTGAAGAGAACGGCGTCACGAGTAAAACTCATTGCGTTGCCATGACTCATGGGCTTTTTCGGGCATCCGACATCGTTTACTAGCCCGAAATCCGGCCTTTACAATTTACGGAAACTATGTTATTTTCCGATTTTAATTCTCGCGTTAATCCTGGGCTTTCTAGGAGCATTTAAGCGTAGTTTGCGTCGAGTGTCACGCGGCGTCACTGGGTCGTGGAATAACAAAGACAGCCAGATGAATAAGACGACGGAAAGCAAACGCCCACGGCAATTGGAACGGCAAAAGGGCAAAAAGAAGGGATGCAACACGAGGACTTCCCAGGAGGTCACCCATCCTAGTACTACTCTCGCCCAAACTCGCTTAACTTCGGAGTTCTGATGGGATCCGGTGCATGTGAGTTGGTATGATCGCATCCGTTAGCTTATAACGCCTAAAAGTATATAAGCCACGTTCGAGCCATGCCATATGACCACGAGTTGGCTATCAAACGCCCCCCCACGTGAAAAGGCATGGCCTGTCAGGCCTTGTCCTCGCCACCCCTACCTTGTGC

>Seq11 [organism= Prunus x yedoensis] B3-1 denotes leaf 1 on branch B3

CAACGGAAAATCGGGTAAACCGCAGATCCGGGCTCCGACCTCGGAATTGGGCCCCTGAACTGCCGATTTGAAGAGAACGGCGTCACGAGTAAAACTCATTGCGTTGCCATGACTCATGGGCTTTTTCGGGCATCCGACATCGTTTACTAGCCCGAAATCCGGCCTTTACAATTTACGGAAACTATGTTATTTTCCGATTTTAATTCTCGCGTTAATCCTGGGCTTTCTCGGAGCATTTAAGCGTAGTTTGCGTCGATTGTCACGCGGCGTCACTGGGTCGTGGAATAACAAAGACAGCCAGATGAATAAGACGACGGAAAGCAAACGCCCACGGCAATTGGAACGGCAAAAGGGCAAAAAGAAGGGATGCAACACGAGGACTTCCCAGGAGGTCACCCATCCTAGTACTACTCTCGCCCAAACTCGCTTAACTTCGGAGTTCTGATGGGATCCGGTGCATGTGAGTTGGTATGATCGCATCCGTTAGCTTATAACGCCTAAAAGTATATAAGCCACGTTCGAGCCATGCCATATGACCACGGGTTGGCTATCAAACGCCCCCCCACGTGAAAAGGCATGGCCTGTCAGGCCTTGTCCTCGCCACCCCTACCTTGTGC

>Seq12 [organism= Prunus x yedoensis] B3-1 denotes leaf 1 on branch B3

GCACAAGGTAGGGGTGGCGAGGACAAGGCCTGACAGGCCATGCCTTTTCACGTGGGGGGGCGTTTGATAGCCAACCCGTGGTCATATGGCATGGCTCGAACGTGGCTTTTATACTTTTAGGCGTTATAAGCTAACGGATGCGATCATACCAACTCACATGCACCGGATCCCATCAGAACTCCGAAGTTAAGCGAGTTTGGGCGAGAGTAGTACTAGGATGGGTGACCTCCTGGGAAGTCCTCGTGTTGCATCCCTTCTTTTTGCCCTTTTGCCGTTCCAATTGCCGTGGGCGTTTGCTTTCCGTCGTCTTATTCATCCGGCTGTCTTTGTTATTCCACGACCCAGTGACGCCGCGTGACAATCGACGCAAACTACGCTTAAATGCTCCGAGAAAGCCCAGGATTAACGCGAGAATTAAAATCGGAAAATAACATAGTTTCCGTAAATTGTAAAGGCCGGATTTCGGGCTAGTAAACGATGTCGGATGCCCGAAAAAGCCCATGAGTCATGGCAACGCAATGAGTTTTACTCGTGACGCCGTTCTCTTCAAATCGGCAGTTCAGGGGCCCAATTCCGAGGTCGGAGCCCGGATCTGCGGTTTACCCGATTTTCCGTTG

>Seq13 [organism= Prunus x yedoensis] B4-1 denotes leaf 1 on branch B4

CAACGGAAAATCGGGTAAACCGCAGATCCGGGCTCCGACCTCGGAATTGGGCCCCTGAACTGCCGATTTGAAGAGAACGGCGTCACGAGTAAAACTCATTGCGTTGCCATGACTCATGGGCTTTTTCGGGCATCCGACATCGTTTACTAGCCCGAAATCCGGCCTTTACAATTTACGGAAACTATGTTATTTTCCGATTTTAATTCTCGCGTTAATCCTGGGCTTTCTCGGAGCATTTAAGCGTAGTTTGCGTCGATTGTCACGCGGCGTCACTGGGTCGTGGAATAACAAAGACAGCCAGATGAATAAGACGACGGAAAGCAAACGCCCACGGCAATTGGAACGGCAAAAGGGCAAAAAGAAGGGATGCAACACGAGGACTTCCCAGGAGGTCACCCATCCTAGTACTACTCTCGCCCAAACTCGCTTAACTTCGGAGTTCTGATGGGATCCGGTGCATGTGAGTTGGTATGATCGCATCCGTTAGCTTATAACGCCTAAAAGTATATAAGCCACGTTCGAGCCATGCCATATGACCACGGGTTGGCTATCAAACGCCCCCCCACGTGAAAAGGCATGGCCTGTCAGGCCTTGTCCTCGCCACCCCTACCTTGTGC

>Seq14 [organism= Prunus x yedoensis] B4-1 denotes leaf 1 on branch B4

GCACAAGGTAGGGGTGGCGAGGACAAGGCCTGACAGGCCATGCCTTTTCACGTGGGGGGGCGTTTGATAGCCAACCCGTGGTCATATGGCATGGCTCGAACGTGGCTTATATACTTTTAGGCGTTATAAGCTAACGGATGCGATCATACCAACTCACATGCACCGGATCCCATCAGAACTCCGAAGTTAAGCGAGTTTGGGCGAGAGTAGTACTAGGATGGGTGACCTCCTGGGAAGTCCTCGTGTTGCATCCCTTCTTTTTGCCCTTTTGCCGTTCCAATTGCCGTGGGCGTTTGCTTTCCGTCGTCTTATTCATCTGGCTGTCTTTGTTATTCCACGACCCAGTGACGCCGCGTGACAATCGACGCAAACTACGCTTAAATGCTCCGAGAAAGCCCAGGATTAACGCGAGAATTAAAATCGGAAAATAACATAGTTTCCGTAAATTGTAAAGGCCGGATTTCGGGCTAGTAAACGATGTCGGATGCCCGAAAAAGCCCATGAGTCATGGCAACGCAATGAGTTTTACTCGTGACGCCGTTCTCTTCAAATCGGCAGTTCAGGGGCCCAATTCCGAGGTCGGAGCCCGGATCTGCGGTTTACCCGATTTTCCGTTG

>Seq15 [organism= Prunus x yedoensis] B4-1 denotes leaf 1 on branch B4

GCACAAGGTAGGGGTGGCGAGGACAAGGCCTGACAGGCCATGCCTTTCCACGTGGGGGGGCGTTTGATAGCCTACCCGTGGTCATATGGCATGGCTCGAACGTGGCTTATATACTTTTAGGCGTTATAAGCTAACGGATGCGATCATACCAACTCACATGCACCGGATCCCATCAGAACTCCGAAGTTAAGCGAGTTTGGGCGAGAGTAGTACTAGGATGGGTGACCTCCTGGGAAGTCCTCGTGTTGCATCCCTTCTTTTTGCCCTTTTGCCGTTCCAATTGCCGTGGGCGTTTGCTTTCCGTCGTCTTATTCATCTGGCTGTCTTTGTTATTCCACGACCCAGTGACGCCGCGTGACAATCGACGCAAACTACGCTTAAATGCTCCGAGAAAGCCCAGGATTAACGCGAGAATTAAAATCGGAAAATAACATAGTTTCCGTAAATTGTAAAGGCCGGATTTCGGGCTAGTAAACGATGTCGGATGCCCGAAAAAGCCCATGAGTCATGGCAACGCAATGAGTTTTACTCGTGACGCCGTTCTCTTCAAATCGGCAGTTCAGGGGCCCAATTCCGAGGTCGGAGCCCGGATCTGCGGTTTACCCGATTTTCCGTTG

>Seq16 [organism= Prunus x yedoensis] B4-1 denotes leaf 1 on branch B4

GCACAAGGTAGGGGTGGCGAGGACAAGGCCTGACAGGCCATGCCTTTTCACGTGGGGGGGCGTTTGATAGCCAACCCGTGGTCATATGGCATGGCTCGAACGTGGCTTATATACTTTTAGGCGTTATAAGCTAACGGATGCGATCATACCAACTCACATGCACCGGATCCCATCAGAACTCCGAAGTTAAGCGAGTTTGGGTGAGAGTAGTACTAGGATGGGTGACCTCCTGGGAAGTCCTCGTGTTGCATCCCTTCTTTTCGCCCTTTTGCCGTTCCAATTGCCGTGGGCGTTTGCTTTCCGTCGTCTTATTCATCTGGCTGTCTTTGTTATTCCACGACCCAGTGACGCCGCGTGACACTCGACGCAAACTACGCTTAAATGCTCCTAGAAAGCCCAGGATTAACGCGAGAATTAAAATCGGAAAATAACATAGTTTCCGTAAATTGTAAAGGCCGGATTTCGGGCTAGTAAACGATGTCGGATGCCCGAAAAAGCCCATGAGTCATGGCAACGCAATGAGTTTTACTCGTGACGCCGTTCTCTTCAAATCGGCAGTTCAGGGGCCCAATTCCGAGGTCGGAGCCCGGATCTGCGGTTTACCCGATTTTCCGTTG

>Seq17 [organism= Prunus x yedoensis] B4-1 denotes leaf 1 on branch B4

GCACAAGGTAGGGGTGGCGAGGACAAGGCCTGACAGGCCATGCCTTTTCACGTGGGGGGGCGTTTGATGGCCAACCCGTGGTCATATGGCATGGCTCGAACGTGGCTTATATACTTTTAGGCGTTATAAGCTAACGGATGCGATCATACCAACTCACATGCACCGGATCCCATCAGAACTCCGAAGTTAAGCGAGTTTGGGCGAGAGTAGTACTAGGATGGGTGACCTCCTGGGAAGTCCTCGTGTTGCATCCCTTCTTTTTGCCCTTTTGCCGTTCCAATTGCCGTGGGCGTTTGCTTTCCGTCGTCTTATTCATCTGGCTGTCTTTGTTATTCCACGACCCAGTGACGCCGCGTGACAATCGACGCAAACTACGCTTAAATGCTCCGAGAAAGCCCAGGATTAACGCGAGAATTAAAATCGGAAAATAACATAGTTTCCGTAAATTGTAAAGGCCGGATTTCGGGCTAGTAAACGATGTCGGATGCCCGAAAAAGCCCATGAGTCATGGCAACGCAATGAGTTTTACTCGTGACGCCGTTCTCTTCAAATCGGCAGTTCAGGGGCCCAATTCCGAGGTCGGAGCCCGGATCTGCGGTTTACCCGATTTTCCGTTG

>Seq18 [organism= Prunus x yedoensis] B4-1 denotes leaf 1 on branch B4

CAACGGAAAATCGGGTAAACCGCAGATCCGGGCTCCGACCTCGGAATTGGGCCCCTGAGCCGTCGATTTGAAGAGAACGGCGTCACGAGTAAAACTCATTGCGTTGCCATGACTCACGGGCTTTTTCGGGCATCCGGCATCGTTTACTAGCCCGAAATCCGGCCTTTACAATTTACGGAAACTATGTTATTTTCCGATTTTAATTCTCGCGTTAATCCTGGGCTTTCTCGGAGCATTTAAGCGTAGTTTGCGTCGATTGTCACGCGGCGTCACTGGGTCGTGGAATAACAAAGACAGCCAGATGAATAAGACGACGGAAAGCAAACGCCCACGGCAATTGGAACGGCAAAAGGGCAAAAGGAAGGGATGCAATACGAGGACTTCCCAGGAGGTCACCCATCCTAGTACTACTCTCGCCCAAACTCGCTTAACTTCGGAGTTCTGATGGGATCCGGTGCATGTGAGTTGGTATGATCGCATCCGTTAGCTTATAACGCCTAAAAGTATATAAGCCACGTTCGAGCCATGCCATATGACCACGGGTTGGCTATCAAACGCCCCCCCACGTGAAAAGGCATGGCCTGTCAGGCCTTGTCCTCGCCACCCCTACCTTGTGC

>Seq19 [organism= Prunus x yedoensis] B4-1 denotes leaf 1 on branch B4

GCACAAGGTAGGGGTGGCGAGGACAAGGCCTGACAGGCCATGCCTTTTCACGTGGGGGGGCGTTTGATAGCCAACCCGTGGTCATATGGCATGGCTCGAACGTGGCTTATATACTTTTAGGCGTTATAAGCTAACGGATGCGATCATACCAACTCACATGCACCGGATCCCATCAGAACTCCGAAGTTAAGCGAGTTTGGGCGAGAGTAGTACTAGGATGGGTGACCTCCTGGGAAGTCCTCGTGTTGCATCCCTTCTTTTTGCCCTTTTGCCGTTCCAATTGCCGTGGGCGTTTGCTTTCCGTCGTCTTATTCATCTGGCTGTCTTTGTTATTCCACGACCCAGTGACGCCGCGTGACACTCGACGCAAACTACGCTTAAATGCTCCTAGAAAGCCCAGGATTAACGCGAGAATTAAAATCGGAAAATAACATAGTTTCCGTAAATTGTAAAGGCCGGATTTCGGGCTAGTAAACGATGTCGGATGCCCGAAAAAGCCCATGAGTCATGGCAACGCAATGAGTTTTACTCGTGACGCCGTTCTCTTCAAATCGGCAGTTCAGGGGCCCAATTCCGAGGTCGGAGCCCGGATCTGCGGTTTACCCGATTTTCCGTTG

>Seq20 [organism= Prunus x yedoensis] B5-1 denotes leaf 1 on branch B5

GCACAAGGTAGGGGTGGCGAGGACAAGGCCTGACAGGCCATGCCTTTTCACGTGGGGGGGCGTTTGATAGCCAACCCGTGGTCATATGGCATGGCTCGAACGTGGCTTATATACTTTTAGGCGTTATAAGCTAACGGATGCGATCATACCAACTCACATGCACCGGATCCCATCAGAACTCCGAAGTTAAGCGAGTTTGGGCGAGAGTAGTACTAGGATGGGTGACCTCCTGGGAAGTCCTCGTGTTGCATCCCTTCTTTTTGCCCTTTTGCCGTTCCAATTGCCGTGGGCGTTTGCTTTCCGTCGTCTTATTCATCTGGCTGTCTTTGTTATTCCACGACCCAGTGACGCCGCGTGACAATCGACGCAAACTACGCTTAAATGCTCCGAGAAAGCCCAGGATTAACGCGAGAATTAAAATCGGAAAATAACATAGTTTCCGTAAATTGTAAAGGCCGGATTTCGGGCTAGTAAACGATGTCGGATGCCCGAAAAAGCCCATGAGTCATGGCAACGCAATGAGTTTTACTCGTGACGCCGTTCTCTTCAAATCGGCAGTTCAGGGGCCCAATTCCGAGGTCGGAGCCCGGATCTGCGGTTTACCCGATTTTCCGTTG

>Seq21 [organism= Prunus x yedoensis] B5-1 denotes leaf 1 on branch B5

GCACAAGGTAGAGGTGGCGAGGACAAGGCCTGACAGGCCATGCCTTTTCACGTGGGGGGGCGCTTGATAGCCAACCCGTGGTCATATGGCATGGCTCGAACGTGGCTTATATACTTTTAGGCGTTATAAGCTAACGGATGCGATCATACCAACTCACATGCACCGGATCCCATCAGAACTCCGAAGTTAAGCGAGTTTGGGCGAGAGTAGTACTAGGATGGGTGACCTCCTGGGAAGTCCTCGTGTTGCATCCCTTCTTTTTGCCCTTTTGCCGTTCCAATTGCCGTGGGCGTTTGCTTTCCGTCGTCTTATTCATCTGGCTGTCTTTGTTATTCCACGACCCAGTGACGCCGCGTGACAATCGACGCAAACTACGCTTAAATGCTCCGAGAAAGCCCAGGATTAACGCGAGAATTAAAATCGGAAAATAACATAGTTTCCGTAAATTGTAAAGGCCGGATTTCGGGCTAGTAAACGATGTCGGATGCCCGAAAAAGCCCATGAGTCATGGCAACGCAATGAGTTTTACTCGTGACGCCGTTCTCTTCAAATCGGCAGTTCAGGGGCCCAATTCCGAGGTCGGAGCCCGGATCTGCGGTTTACCCGATTTTCCGTTG

>Seq22 [organism= Prunus x yedoensis] B5-1 denotes leaf 1 on branch B5

CAACGGAAAATCGGGTAAACCGCAGATCCGGGCTCCGACCTCGGAATTGGGCCCCTGAACTGCCGATTTGAAGAGAACGGCGTCACGAGTAAAACTCATTGCGTTGCCATGACTCATGGGCTTTTTCGGGCATCCGACATCGTTTACTAGCCCGAAATCCGGCCTTTACAATTTACGGAAACTATGTTATTTTCCGATTTTAATTCTCGCGTTAATCCTGGGCTTTCTCGGAGCATTTAAGCGTAGTTTGCGTCGATTGTCACGCGGCGTCACTGGGTCGTGGAATAACAAAGACAGCCAGATGAATAAGACGACGGAAAGCAAACGCCCACGGCAATTGGAACGGCAAAAGGGCAAAAAGAAGGGATGCAACACGAGGACTTCCCAGGAGGTCACCCATCCTAGTACTACTCTCGCCCAAACTCGCTTAACTTCGGAGTTCTGATGGGATCCGGTGCATGTGAGTTGGTATGATCGCATCCGTTAGCTTATAACGCCTAAAAGTATATAAGCCACGTTCGAGCCATGCCATATGACCACGGGTTGGCTATCAAGCGCCCCCCCACGTGAAAAGGCATGGCCTGTCAGGCCTTGTCCTCGCCACCTCTACCTTGTGC

>Seq23 [organism= Fagus crenata] B2-1 denotes leaf 1 on branch 2 of tree B

TGCAAGTGGGGCCGGGATTGTACTCTTTAGAAATGATGGAGAGGCTATACCAAAGTCTTTTAAGCTTGATTTTCCATGCTCTAACAACGTCGCGGAGTACGAAGCTTATCTTACTGGACTGGTAGTAGCATGGGAGATGGGGATCAAACACTTGAAAGTTGTCGGAGACTCCAATCTGATTGTTTGCCAGGCTCGTGGAGAATTTTCACTTAAGGAACCATCCTTAGCACCGTACCGAGCTCTAGCACAGAAGTTGGAAGAGAAATTTGTCACCTTTGAGATTGAGCATGCCCAAAGAAATGAGAACCGCTATGCGGATGCACTCGCAACTTTGGGGTCTCAAATGGCCTTTGAAGGACAGAAAATTGACATCACCATCAACAAAAAAGTGAGGCCTATCACTGAGTTGTTGAAAAAAGAGTTTGAGGAGTTGTCTCTCAATGAAGAAGACTGGAGAATGCCACTTAAAGCCAAGCTCGTGTCTCCA

>Seq24 [organism= Fagus crenata] B2-1 denotes leaf 1 on branch 2 of tree B

ACTCGTTGATCATGCAAGGTCACTTGCGATTGAGCCAAAGCCTGAAAATGTGAGTGAATTTCAATCTTTTCCAGGAGAAGGCATTCATGGCAACATTGATGGTAATGATATTTACATTGGAAACAGAAAAATTGCTTTGAGAGCTGGGTGTAGAACAGGTGAGAGCCTTCACTTTCTTTCTTTCTTTTTTTTTAACTCAAAACTATATACTAAACACTACACTTTCATATTAAATATGTTGTACATTAATCCATGCTATCATTTTTTTTTTTACAAAGAATTTTTGAAAGATTTAGCTGTACGTTTCCAAATTTTAATTTCATTTTCCTCTAGCAATAGAATTGGCTTTGCGTTACATTTTCATGGTTCTAATCCTATGATATTCAACTCTTTTAGTCCCCACCTTAGAGGGTGATGTGGAGGGAGGAAAGACCATTGGTTATATATACTCTGGAGCAACCCCAGCTGGAATTTTCAGTCTCTCTGATGCCTGTCGATCTGGGGTTCAGGATGCGATCAGGGAGCTGAAGTTGTTGGGTATTAAGACTGCTATGCTTACCGGAGACAGTCAT

>Seq25 [organism= Fagus crenata] B2-1 denotes leaf 1 on branch 2 of tree B

TGCATCAAGTGGGGCTAGGATTGTACTTTTTAGAAATGATGGAGAGGCTATACCAAAGTCTTTTAAGCTTGATTTTCCATGTTCTAACAACGTCGCGGAGTATGAAGCTTATCTTACTGGACTGGCAGTAGCATGGGAGATGGGGATCAAACACTTGAAAGTTGTCGGAGACTCCAATCTGATTGTTTGCCAGGCTCGTGGAGAATTTTCACTTAAGGAACCATCCTTAGCACCATACCGAGCTCTAGCACAGAAGTTGGAAGAGAAATTTGTCACCTTTGAGATCGAGCATGCCCAAAGAAACGAGAACCGCTATGCGGACGCACTTGCAACTTTGGGGTCTCAAATGGCCTTTGAAGGACAGAAAATTGACATCACCATCAACAAAAAAGTGAGACCTATCACTGAGTTGTTGAAAGAAGAGTTTGAAGAGTTATCTCTCAATGAAGAGGACTGGAGAATGCCACTTAAAGCCAAGCTCGTGTCTCCA

>Seq26 [organism= Fagus crenata] B2-1 denotes leaf 1 on branch 2 of tree B

TGCTAACTCGGCGGGCTTGAGAATCTTTGTCTAGTGTATTACTGCCACTAGGTTGAAGATCACTCCCACTATCTTCAGCGATTACAGAACGAGGAACTAATCCCCCACCCTCGCTTGAAGATGGTAAGGTACCTTCATCTTCCTCTAACTCATAGAGGTCAAGATCTCTGTTAGCATCGCCAATGCTAGGGAAATCAGTCTCGATGAAATCGATATCGCGCGACTCTATTTCAGTCATTCCTCCATTGGGATGTTCACCATACATGACATATCCTTTCGAGCTATCAGAATATCTTATGAAGATATGCTTCCTAGCTTTAGGGCCCAATTTTCCATACTTATGGGCGGTGCTGTGTACAAAACCAGCAGATCCCCAAGGGCGTAAATGCTCCAAATTGGGCTTTTCGCCTTTCCAAAGTTCATATGGGGTGGAGGAAACTGACTGAGAAGGCACACGGTTAAGTATGTAG

>Seq27 [organism= Fagus crenata] B2-1 denotes leaf 1 on branch 2 of tree B

ATCGAGTGGAGCTAGGATTGTACTCTTTAGAAATGATGCAGAGGCTATACCAAAGTCTTTTAAGCTTGATTTCCCATGCTCTAACAACGTCGCGGAGTACGAAGCTTATCTTACTGGACTGGCAGTAGCATGGGAGATGGGGATCAAGCACTTGAAAGTTGTCGGAGACTCCAATCTGATTGTTTGCCAGGCTCGTGGAGAATTTTCACTTAAGGAACCATCCTTAGCACCATACCGAGCTCTAGCACAGAAGTTGGAAGAGAAATTTGTCACCTTTGAGATCGAGCATGCCCAAAGAAACAAGAACCGCTATGCGGACGCACTTGCAACTTTGGGGTCTCAAATGGCCTTTGAAGGAGAGAAAATTGACATCATCATCAACAAAAAAGTGAGGCCTATCACTGAGTTGTTGAAAAAAGAGTTTGAAGAGTTACCTCTCAATGAAGAAGACTGGAGAATGCCGCTTAAAGCCAAGCTCGTGTCTCCA

>Seq28 [organism= Fagus crenata] B2-2 denotes leaf 2 on branch 2 of tree B

TGCTGCATCAAGTGGGGCTGGGATTGTACTTTTTAGAAATGATGGAGAGGCTATACCAAAGTCTTTTAAGCTTGATTTTCCATGCTCTAACAACGTCGTGGAGTACGAAGCTTATCTTACTGGACTGGCAGTAGCATGGGAGATGGGGATCAAGCACTTGAAAGTGGTCGGAGACTCCAATCTGATTGTTTGCCAGGCTCGTGGAGAATTTTCACTTAAGGAACCATCCTTGGCACCATACCGAGCTCTAGCACAGAAGTTGGAAGAGAAATTTGTCACCTTTGAGATCGAGCATGCCCAAAGAAACGAGAACCGCTATGCGGATGCACTCTCAACTTTGGGGTCTCAAATGGCCTTTGAAGGACAGAAAATTGACATCACCATCAACAAAAAGGTGAGGCCTATCACTGAGTTGTTGAAAGAAGAGTTTGAAGAGTTATCTCTCAATGAAGAAGACTGGAGAATGCCACTTAAAGCCAAGCTCGTGTCTCCA

>Seq29 [organism= Fagus crenata] B2-2 denotes leaf 2 on branch 2 of tree B

ATCAAGTGGGGCTGGGATTGTACTTTTTAGAAATGATGGAGAGGCTATACCAAAGTCTTTTAAGCTTGATTTTCCATGCTCTAACAACGTCGCGGAGTACGAAGCTTATCTTACTGGACTGGCAGTAGCATGGGAGATGGGGATCAAACACTTGAAAGTTGTCGGAGACTCCAATCTGATTGTTTGCCAGGCTCGTGGAGAATTTTCACTTAAGGAACCATCCTTAGCACCATACCGAGCTCTAGCACAGAAGTTGGAAGAGAAATTTGTCACCTTTGAGATCGAGCATGCCCAAAGAAACGAGAACCGCTATGCGGACGCACTTGCAACTTTGGGGTCTCAAATGGCCTTTGAAGGACAGAAAATTGACATCACCATCAACAAAAAAGTGAGACCTATCACTGAGTTGTTGAAAGAAGAGTTTGAAGAGTTATCTCTCAATGAAGAGGACTGGAGAATGCCACTTAAAGCCAAGCTCGTGTCTCCA

>Seq30 [organism= Fagus crenata] B2-2 denotes leaf 2 on branch 2 of tree B

TGCTGCAGTATTTGTTGAATCAGAAGAAAAATCCAACAAATAGAGCCCATTATGTTGTTTACCCATACCAATCATCCTCCAATGCATGAGGTCCTGTATAAAACAGTAAGTGGACAAGAAGAAAATGCAACAATGGAGAGATGAAGTAAGTTTGGAGACAGAAATCATATTGAAATCAAAAGATGGTACACATAACACATCTGTGAGGAGCAAGTTAGGAGTAATTTGGACAGAACCAATGTGTGTGACCAAAACAGACTGTCCATTAGGTAAATTGACACTAATGTTGTCCACACAATGCATAATAGTATAAAATTGTGTGGTGATGACCATATGGTCAGTAGCACCAGTGTCAATAACCCATTGAGCAGGAGAAAAATGTGGCTCGACAGTAAACTTAGCAGAAAAGACAGAATGGTCCATATTAGGCTTGCTAAAAGTTGAAAGACAAGTTGGTTTACCTGCCATGTTGGAGTGAGGTTGTGTTACAGAGATTGAGGAGATTGAG

>Seq31 [organism= Fagus crenata] B2-2 denotes leaf 2 on branch 2 of tree B

TGGATAAGTGTTTCGATTCAAGAGAGGCAGATTTATTTTCTGTGTGTTTGCCTTGCCAATTGGTCAAGGTGAATTGTCTATTTATAAGAGATTACAAAATAAGATATATACAAGAAGAGAGATTGATAATCATAACAACTCTAACAATATAAACTCCTAGTTTACAGGTAACTAATCTTGATCTTGATCTTTATCTTCTTGAACTGTTTTAGTTGGACTCAATTGTGGAGTATCCTTAACACTCCCCCTCAAGTCCAACGGTATGGGAAGAACGTTGAGCTTGGAACGGAACAAGACAAACCGAGGAGAGGACAGGGGTTTTGTCAAAAGATCAGCAAGTTGGTCATTTGTGGAGACAAACCGAACCAAAAGTGTTTTAGAGGCCACCATGTCACGAACAAAGTGAAAATCTATCTCAATGTGCTTGGTTCGGGCATGGAAAACAGGATTTGCGGAGAGATAAGTTGCGCCAATATTGTCACACCAAAGAACTGTAGAGGATGGAGGTGGTTGGCCAAGTTCAGTGAGAAGAGAATGAAGCCATTGGACC

>Seq32 [organism= Fagus crenata] B2-3 denotes leaf 3 on branch 2 of tree B

CATTGCTTATGGTCTTGACAAGAAGGCAACCAGTGTTGGCGGGAAGAATGTTTTGATTTTTGATCTTGGTGGTGGTACTTTTGATGTCTCACTTCTTACCATTGAAGAGGGTATCTTTGAAGTAAAGGCTACAGCTGGTGACACTCATCTTGGTGGGGAGGACTTCGATAACAGAATGGTGAACCACTTTGTTCAGGAGTTCAAGAGGAAGAACAAGAAGGATATTAGTGGGAACCCCAGAGCCCTTAGGAGGTTGAGGACCTCATGTGAGAGGGCGAAGAGGACACTATCATCCACTGCTCAGACCACCATTGAGATTGACTCATTGTATGAAGGCATTGACTCCTATTCAACCATTACTCGTGCTAGGTTTGAGGAGCTCAACATGGATCTCTTTAGAAAGTGTATGGAGCCTGTGGAGAAGTGTTTGAGGGATGCAAAGATGGACAAGAGCACCATCCATGATGCTGTCCTTGTTGGGGGTTCTACCAGGATTCCCAAGGT

>Seq33 [organism= Fagus crenata] B2-3 denotes leaf 3 on branch 2 of tree B

TGGAGACACGAGCTTGGCTTTAAGTGGCATTCTCCAGTCTTCTTCATTGAGAGATAACTCTTCAAACTCTTCTTTCAACAACTCAGTGATAGGCCTCACCTTTTTGTTGATGGTGATGTCAATTTTCTGTCCTTCAAAGGCCATTTGAGACCCCAAAGTTGAGAGTGCATCCGCATAGCGGTTCTCGTTTCTTTGGGCATGCTCGATCTCAAAGGTGACAAATTTCTCTTCCAACTTCTGTGCTAGAGCTCGGTATGGTGCCAAGGATGGTTCCTTAAGTGAAAATTCTCCACGAGCCTGGCAAACAATCAGATTGGAGTCTCCGACCACTTTCAAGTGCTTGATCCCCATCTCCCATGCTACTGCCAGTCCAGTGAGATAAGCTTCGTACTCCACGACGTTGTTGGAGCATGGAAAATCAAGCTTAAAAGACTTTGGTATAGCCTCTCCATCATTTCTAAAAAGTACAATCCCAGCCCCACTTGAT

>Seq34 [organism= Fagus crenata] B2-3 denotes leaf 3 on branch 2 of tree B

CTACATACTTAACTGTGTGCCTTCTCACTCTATTTCCTCCACCTCATACAAGCTTTGGAGAGGCGAAAAGCCCAATTTGGAGTATTTATGCCCTTGAAGATCTGTTGGTTTTGTACACAACACCACCCATAAGTATGGAAAATTGGGCCCTAGAGCTAGGAAGCATATCTTCATAAGATATTCTAATAACTCGAAAGGATATGTCATGCATGGTGAACATCCCAATGGAGGAATGACTGAAATAGAGTCGTGCGATATCGATTTCATCGAGACTGATTTCCCTAACATTGGTGATGCTAACAAAGATCTTGGCCTCTATGAGTTAAAGGAAGATGAAGGCACCTTACCATCTTCAAGCGAGGGTGGGGGATTAATTCCTCATCTTGTAATCGCTGAAGATAGTGGGAGTGGTCTTCAACCTAGTGAGAGTATTACACTAGCTCAAGAATCTCAAGTGCGCCGAGTTA

>Seq35 [organism= Fagus crenata] B2-3 denotes leaf 3 on branch 2 of tree B

ATCAAGTGGGGCTGGGATTGTACTTTTTAGAAATGATGGAGAGGCTATACCAAAGTCTTTTAAGCTTGATTTTCCATGCTCCAACAACGTCGTGGAGTACGAAGCTTATCTCACTGGACTGGCAGTAGCATGGGAGATGGGGATCAAGCACTTGAAAGTGGTCGGAGACTCCAATCTGATTGTTTGCCAGGCTCGTGGAGAATTTTCACTTAAGGAACCATCCTTGGCACCATACCGAGCTCTAGCACAGAAGTTGGAAGAGAAATTTGTCACCTTTGAGATCGAGCATGCCCAAAGAAACGAGAACCGCTATGCGGATGCACTCTCAACTTTGGGGTCTCAAATGGCCTTTGAAGGACAGAAAATTGACATCACCATCAACAAAAAGGTGAGGCCTATCACTGAGTTGTTGAAAGAAGAGTTTGAAGAGTTATCTCTCAATGAAGAAGACTGGAGAATGCCACTTAAAGCCAAGCTCGTGTCTCCA

>Seq36 [organism= Fagus crenata] B2-3 denotes leaf 3 on branch 2 of tree B

ATCAAGTGGGGCTGGGATTGTACTTTTTAGAAATGATGGAGAGGCTATACCAAAGTCTTTTAAGCTTGATTTTCCATGCTCCAACAACGTCGTGGAGTACGAAGCTTATCTCACTGGACTGGCAGTAGCATGGGAGATGGGGATCAAGCACTTGAAAGTGGTCGGAGACTCCAATCTGATTGTTTGCCAGGCTCGTGGAGAATTTTCACTTAAGGAACCATCCTTGGCACCATACCGAGCTCTAGCACAGAAGTTGGAAGAGAAATTTGTCACCTTTGAGATCGAGCATGCCCAAAGAAACGAGAACCGCTATGCGGATGCACTCTCAACTTTGGGGTCTCAAATGGCCTTTGAAGGACAGAAAATTGACATCACCATCAACAAAAAGGTGAGGCCTATCACTGAGTTGTTGAAAGAAGAGTTTGAAGAGTTATCTCTCAATGAAGAAGACTGGAGAATGCCACTTAAAGCCAAGCTCGTGTCTCCA

>Seq37 [organism= Fagus crenata] B2-3 denotes leaf 3 on branch 2 of tree B

TGGAGACACGAGCTTGGCTTTAAGTGGCATTCTCCAGTCTTCTTCATTGAGAGATAACTCTTCAAACTCTTCTTTCAACAACTCAGTGATAGGCCTCACCTTTTTGTTGATGGTGATGTCAATTTTCTGTCCTTCAAAGGCCATTTGAGACCCCAAAGTTGAGAGTGCATCCGCATAGCGGTTCTCGTTTCTTTGGGCATGCTCGATCTCAAAGGTGACAAATTTCTCTTCCAACTTCTGTGCTAGAGCTCGGTATGGTGCCAAGGATGGTTCCTTAAGTGAAAATTCTCCACGAGCCTGGCAAACAATCAGATTGGAGTCTCCGACCACTTTCAAGTGCTTGATCCCCATCTCCCATGCTACTGCCAGTCCAGTGAGATAAGCTTCGTACTCCACGACGTTGTTGGAGCATGGAAAATCAAGCTTAAAAGACTTTGGTATAGCCTCTCCATCATTTCTAAAAAGTACAATCCCAGCCCCACTTGAT

>Seq38 [organism= Fagus crenata] B2-3 denotes leaf 3 on branch 2 of tree B

TGGTAAAAGTCTAAAAAAGTCAACACTGGTAAAAGTCAATGTTGCTGGTACAAAAGTCTATCATAAAAAATTCAACGACGACTGCTGAAAGTTAATGTTGCTAGTTAACTTGTTAGAAAACTCAACCGTAAAAAATAAATGACAGCTGTCGAAAGTCAATTGTAAAGAGTCAACGTAACGGTCAACTCGGCAGAAAGTCAACGAGAAGTCAATGGTGATGACTTGTTAGCTAACGTGGCACTAGGGTTGATGTGGCGCACTTGGAGGTTGACACGTGGCACAGTCTAATAGGGTAGACGTGTGTGGCGCGTGAAATGGCATGACGGCACACCGGTACTTTGGGTGGCGTATGAGGGCGCATCCGGCGGTGGATTGGGCCGATTTTTGCACAGTTGTGTCGAGCGTCTCGAGATCTTTCCACCGGTAGTGGAGTTGTCTTGATCGGAGTCATGTAGTGGCAATTCTGAGGGTGGTAGCGAGTCTTTTTGGCAGGGATTAGCTTTG

>Seq39 [organism= Fagus crenata] B3-1 denotes leaf 1 on branch 3 of tree B

TGCAAAGAGACTTACTGATTCCCTCATGGGATGGTTAGTTAATCAGCTTCATGCATTTATAACCACATGCCATGTTACATTGTCTTGTACTCCTAGGACCATTGACAGTCTTCTTGCTCCTATGAATTTTGAATGTAATGTTAATTCTATTTAGGTACATTGAACCATTGATGTTTGGAGAATATCCAAAAAGCATGAAACTTTTGGTGAAGGAGAGGCTACCCATTCTTTCTGAAGAAGAGAAAAAGATGATCACAGGAACCCTTGATTTCATCGGTCTCAATTATTATACTTTACGTTTTGCTAAAGCCACTGCGCCAGCTCAATATCCCCGCCATATATTCGATGCATTAGCATATG

>Seq40 [organism= Fagus crenata] B3-1 denotes leaf 1 on branch 3 of tree B

AAAGAGACTTACTGATTCCCTCATGGGATGGTTAGTTAATCAGCTTCATGCATTTATAACCACATGCCATGTTACATTGTCTTGTACTCCTAGGACCATTGACAGTCTTCTTGCTCCTATGAATTTTGAATGTAATGTTAATTCTATTTAGGTACATTGAACCATTGATGTTTGGAGAATATCCAAAAAGCATGAAACTTTTGGTGAAGGAGAGGCTACCCATTCTTTCTGAAGAAGAGAAAAAGATGATCACAGGAACCCTTGATTTCATCGGTCTCAATTATTATACTTTACGTTTTGCTAAAGCCACTGCGCCAGCTCAATATCCCCGCCATATATTCGATGCATTAGCATATG

>Seq41 [organism= Fagus crenata] B3-1 denotes leaf 1 on branch 3 of tree B

TGCATCAAGTGGGGCTGGGATTGTACTCTTTAGAAATGATGGAGAGGCTATACCAAAGTCTTTTAAGCTTGATTTTCCATGCTCTAACAACGTCGCGGAGTACGAAGCTTATCTTACTGGACTGGCAGTAGCATGGGAGATGGGGATCAAGCACTTGAAAGTTGTCGGAGACTCCAATCTGATTGTTTACCAGGCTCGTGGAGAATTTTCACTTAAGGAACCATCCTTAGCACCGTACCGAGCTTTAGCATAGAAGTTGGAAGAGAAATTTGTCACCTTTGAGATCGAGCATGCCCAAAGAAACGAAAACCGCTATGCGGATGCACTCGCAACTTTGGGGTCTCAAATGGCCTTTGAAGGACAGAAAATTGACATCACCATCAACAAAAAAGTGAGGCCTATCACTGAGTTGTTGAAAAAAGAGTTTGAAGAGTTATCTCTCAATGAAGAAGACTGGAGAATGCCACTTAAAGCCAAGCTCGTGTCTCCA

>Seq42 [organism= Fagus crenata] B3-1 denotes leaf 1 on branch 3 of tree B

CTCCATCAAGTTATGACACTCTCGTCTAGGTCATGTTTCTCTTCCTTGTATTAAAACCTTAATATCTAGGGGTCTATTAGGTTCAGTTTCTTCTAGTCCATTTGATTGTATGCCATGTCAGCTCGGCAAACAACATGCTTTACCTTTTAATAATAGTGAGTCTATTGCTTCTGCAACTTTTGATTTTATTCATTCAGATGTATGGGGGCCCTCTCCTGTTTCCACAGTGGGGTGATTAAGATATTTTGTTATTTTTGTCGATGATTTTTCTCGTTACACTTGGATTTATTTAATGAAAAATCATTCTGAAGTTCTCACCATATATCGTGATTTTGCTAAAATGATTCAGACTTAGTATTCTAAAGCTATTAAAGTATTTCGATCTGACAATGCACGAGAGTATAGACAAACTGATTTTTTTACTATTCTTAAACATTATGGAACTATTTTTCATACCTCGTGTGCTGGCACCTCACAACAAAATGGATAAGCTGAACATAAACTATGTCACATCCTAGATACTGTTAGGGCTCTCACCAAT

>Seq43 [organism= Fagus crenata] B3-1 denotes leaf 1 on branch 3 of tree B

CCAGTGAAGGGACACGTCGCCAGCAAGACGCCACCGCGAGACTGCCACCCACCCACCCCACTGGCAAGGAGGTATCTAGTTTCTTTCTATCTGTAATTTTTCTCCAGGAAATCCTAATTACTAGCTTTTGTTTTTCATTGTGCATACAACAGGTAACCCCTGATAGGTTATACAGCAGCTTTGGTTAATGGGTGGGGACGTTGGGTACATATACTCACCACTTGAAAAAGTTATTACTTATTAGGTTGTCTCGTATGCAATGTCCATAGAAATTTCTCCCTCTTTCTTTCTTATTGCTTTGACTGAGATTTTCTCTCAAGGTGCATTATTTTTCTAAATATCTAATATGAACGAAGATGAAGTTTCAGAATTAAAATCAGAGGACAGAATATTACGGCCACCAGATGAAAGAGAAGTGAACCGGATTAAACCAAAGCTAACTCTTAAAGTTCAACCAGTCTACATGGACCCAAAGCCTGAAAAAGGTAGACAACCAAATAAGGCCCTAACCAACAGCTTATGCTTGAAGATAATAGGGAGGGT

>Seq44 [organism= Fagus crenata] B3-1 denotes leaf 1 on branch 3 of tree B

TGCATCGAGTGGAGCTAGGATTGTACTTTTTAGAAATGATGCAGAGGCTATACCAAAGTCTTTTAAGCTTGATTTCCCATGCTCTAACAACGTCGCGGAGTACGAAGCTTATCTTACTGGACTGGCAGTAGCATGGGAGATGGGGATCAAGCACTTGAAAGTTGTCGGAGACTCCAATCTGATTGTTTGCCAGGCTCGTGGAGAATTTTCACTTAAGGAACCATCCTTAGCACCATACCGAGCTCTAGCACAGAAGTTGGAAGAGAAATTTGTCACCTTTGAGATCGAGCATGCCCAAAGAAACAAGAACCGCTATGCGGACGCACTTGCAACTTTGGGGTCTCAAATGGCCTTTGAAGGAGAGAAAATTGACATCATCATCAACAAAAAGTGAGGCCTATCACTGAGTTGTTGAAAAAAGAGTTTGAAGAGTTACCTCTCAATGAAGAAGACTGGAGAATGCCGCTTAAAGCCAAGCTCGTGTCTCCA

>Seq45 [organism= Fagus crenata] B3-2 denotes leaf 2 on branch 3 of tree B

GTTCTCTGCCCCTCCTACTTCACCTGGCTGCACAGTCTTTGCGATTGAAACGTCTAGTTTCATTACCCAATTTCGTATTGACACTTTTGTAATGGGAGTGGGACAAGATCAGGGTTGGACTAGCCATAAGCCATGCATTGATGGATATAGTTTTAGTACAAAAGCCTTCAGTAATGTCGTTCAAGCAATTTGTTATAATGGGGTTTTGTACTGTGTGGATTCACGTGGAAGAATTGGTACTTTTGATGTGAAGTTTGGGAATTGGAACATTGTTCTTAGCAAGAAGTTTGAGAGGGTTTGCACTAGTCCTTATTTGGTGGAATTCAATGGAGAGATTTTTGCAGCAAAGAGAGGAGCTTATGGGGTTGTTGAAAAGCTTTATAAATTGAAGCTTGAACATGGGGTG

>Seq46 [organism= Fagus crenata] B3-2 denotes leaf 2 on branch 3 of tree B

TGCCTTATGGGACTCGATTCTACGCTCTTTTGGGGTTCCTTGGGTGATGCCTGACAACATTGTAGCTCTTCTTTTCGGCTGGTATAACTGTTTTGGAAAGCACAATTCTAAGGTTTGGAATCTGGTGCCTTTATACTTAATGTGGACTGTTCGGCGTGAGTGTAATAGGCGTACTTTCAACGATGAAGAGCATTCGGAGACCAAACTCACTGAATTATTTTTTCGGCTTCTTTTTCATTGGGCTCGGGCTTAGGGCTTTACCTCCGAGTTAACCCTTGCTGATTTTGTTGTTTCTCTAAGTTTTTCTCAGGATTCGTAATTTAGATCCTTTGTATTTATTCTCCCCTAAGTGCATGCTTTATGCCCTTAGAGGGAGTTTCCATTTCTTCTTAATACAATCCTTATTACTTATCAAAAAAAAAAAAAAAAAAAAAAATCTTTGTATGTGCTTAGGATAATAACCCTTTGAGGTTCAAACTTGCTAGATGGAATAACATTTCCTTTGCCTATCTGATCATTAAGATTGCTATAGCTCTTTGTTTGTCCTATCNATGTAAAAACTCTTTGAAAACAAAACATTTCCTTGCCCCATCCTTTACTTTGT

>Seq47 [organism= Fagus crenata] B3-2 denotes leaf 2 on branch 3 of tree B

TGCATCAAGTGGGGCTGGGATTGTACTTTTTAGAAATGATGGAGAGGCTATACCAAAGTCTTTTAAGCTTGATTTTCCATGCTCTAACAACGTCGTGGAGTACGAAGCTTATCTTACTGGACTGGCAGTAGCATGGGAGATGGGGATCAAGCACTTGAAAGTGGTCGGAGACTCCAATCTGATTGTTTGCCAGGCTCGTGGAGAATTTTCACTTAAGGAACCATCCTTGGCACCATACCGAGCTCTAGCACAGAAGTTGGAAGAGAAATTTGTCACCTTTGAGATCGAGCATGCCCAAAGAAACGAGAACCGCTATGCGGATGCACTCTCAACTTTGGGGTCTCAAATGGCCTTTGAAGGACAGAAAATTGACATCACCATCAACAAAAAGGTGAGGCCTATCACTGAGTTGTTGAAAGAAGAGTTTGAAGAGTTATCTCTCAATGAAGAAGACTGGAGAATGCCACTTAAAGCCAAGCTCGTGTCTCCA

>Seq48 [organism= Fagus crenata] B3-2 denotes leaf 2 on branch 3 of tree B

TGCTTCAAGTGGGGCCGGGATTGTACTTTTTAGAAATGATGGAGAGGCTATACCAAAGTCTTTTAAGCTTGATTTTCCATGCTCTAACAACGTCGCAGAGTACGAAGCTTATCTTACTGGACTGGCAGTAGCATGGGAGATGGGGATCAAGCACTTGAAAGTTGTCGGAGACTCCAATCTGATTGTTTGTTAGGCTCGTGGAGAATATTCACTTAAGGAACCATCCTTAGCACTGTACCGAGCTCTAGCACAGAAGTTGGAAGAGAAATTTGTCACCTTTGAGATCGAGCATGCCCAAAGAAACGAGAACCGCTATGCGGACGCGCTTGCAACTTTGGGGTCTCAAATGGCCTTTGAAGGACAGAAAATTGACATCACCATCAACAAAAAAGTGAGGCCTATCACTGAGTTGTTGAAAGAAGAGTTTGAAGAGTTATCTCTTAATGAAGAAGACTGGAGAATGCCACTTAAGGCCAAGCTCGTGTCTCCA
